# Monocarboxylate Transporter 2 (MCT2) Reduction Is Associated with Increased Lung Tumor Growth and Alterations in the Immune Microenvironment in a Subcutaneous Tumor Model

**DOI:** 10.3390/ijms27177819

**Published:** 2026-08-31

**Authors:** Yen On Chan, Zhuanhong Qiao, Trupti Joshi, David Gozal, Abdelnaby Khalyfa

**Affiliations:** 1 Department of Biomedical Sciences, Joan C. Edwards School of Medicine, Marshall University, Huntington, WV 25755, USAjoshitr@marshall.edu (T.J.); 2 Department of Pediatrics, Joan C. Edwards School of Medicine, Marshall University, Huntington, WV 25755, USA; 3 Department of Electrical Engineering and Computer Science, University of Missouri, Columbia, MO 65201, USA

**Keywords:** MCT2, snRNA-seq, tumors, bulk RNA-seq, cancer, lung cancer cells, macrophages

## Abstract

Monocarboxylate transporter 2 (MCT2; *SLC16A7*) is a high-affinity pyruvate transporter implicated in cancer metabolism. However, its role in lung cancer progression and the tumor microenvironment remains unclear. This study examined the effects of MCT2 reduction on tumor growth and cell-type-specific transcriptional changes within the tumor microenvironment. MCT2 loxP/loxP mice were crossed with mCre-Tg mice, and MCT2 deletion was induced by tamoxifen. Control (CO) mice received vehicle treatment. TC1 cells (100,000 cells/mouse) were injected subcutaneously, and tumors were harvested after 24 days. Single-nucleus RNA sequencing (snRNA-seq) was performed on isolated tumor nuclei (4000 nuclei/sample; *n* = 3 per group) using the 10x Genomics Chromium platform. Data were processed with Cell Ranger v3.0.2 and Seurat v5.2.1, followed by differential expression and pathway enrichment analyses integrated with macrophage bulk RNA-seq data. Tumors in mice with systemic MCT2 reduction grew significantly faster than those in control mice, demonstrating an association between host MCT2 reduction and increased tumor growth. Transcriptomic analysis generated high-quality profiles from 6864 CO and 10,055 KO nuclei. Clustering identified 12 cellular populations and cell types. MCT2 reduction altered pathways involved in glycolysis, the tricarboxylic acid cycle, oxidative phosphorylation, and fatty acid metabolism across multiple populations. Macrophages showed prominent transcriptional changes, including enrichment of MAPK, PI3K-Akt, IgSF-CAM, ECM, and cytokine–cytokine signaling pathways. These findings were supported by macrophage bulk RNA-seq data. Systemic MCT2 reduction was associated with increased tumor growth and broad transcriptional alterations within the tumor micro-environment. Differences in metabolic and immune-related transcriptional programs, particularly in macrophages, identify potential mechanisms associated with tumor progression that warrant further functional investigation.

## 1. Introduction

Lung cancer remains the leading cause of cancer-related mortality worldwide and accounts for approximately 1.8 million deaths annually despite advances in diagnosis and treatment strategies [1,2]. The disease is highly heterogeneous and is strongly influenced by the tumor microenvironment (TME), which plays a central role in tumor initiation, progression, immune evasion, and therapeutic response [3]. Among the major cellular components of the TME are tumor-associated macrophages (TAMs), a highly plastic immune cell population capable of adopting diverse functional states in response to local environmental cues [4]. TAM phenotypes are often broadly categorized into pro-inflammatory M1-like and immunosuppressive M2-like states, although macrophage behavior exists along a dynamic spectrum. Increasing evidence suggests that TAMs actively remodel the tumor immune microenvironment and contribute to tumor progression, metastasis, and resistance to both targeted therapies and immunotherapies [5]. Tumor heterogeneity represents a major challenge in lung cancer management, as both intertumoral and intratumoral variability drive tumor evolution, therapeutic resistance, disease recurrence, and treatment failure. Metabolic reprogramming within the TME is increasingly recognized as a critical regulator of immune cell function, including macrophage polarization and activity. However, the metabolic characteristics and regulatory mechanisms underlying distinct TAM subsets in vivo remain incompletely understood. In particular, the contribution of monocarboxylate transport systems to TAM-associated metabolic heterogeneity in lung cancer has not been fully elucidated.

Monocarboxylate transporter 2 (MCT2), encoded by the *SLC16A7* gene, is a transmembrane transporter involved in the cellular uptake of key metabolic substrates such as pyruvate and lactate [6,7,8,9]. MCT2 belongs to a family of 14 monocarboxylate transporters, among which MCT1-MCT4 are the most extensively studied because of their roles in transporting lactate, pyruvate, and short-chain fatty acids across biological membranes [10]. Dysregulated expressions of several MCT isoforms have been associated with cancer progression and metabolic adaptation, making them potential biomarkers and therapeutic targets [8,11]. Compared with MCT1, MCT2 exhibits higher affinity for pyruvate and lactate, suggesting that it may play an important role in supporting metabolic adaptation under nutrient- and oxygen-limited conditions [8,12,13,14,15,16,17]. Elevated MCT2 expression has been reported in several types of cancer, where it may contribute to metabolic reprogramming and maintenance of oxidative metabolism [18]. In glycolytic tumors, monocarboxylate transporters facilitate lactate transport, regulate intracellular pH, and help maintain a microenvironment that supports tumor survival and invasion [16,19]. Nevertheless, the specific role of MCT2 in shaping the lung tumor microenvironment, particularly in TAM populations, remains poorly defined.

Recent advances in multi-omics technologies have provided powerful tools for characterizing tumor complexity and cellular heterogeneity at high resolution. Integration of single-nucleus RNA sequencing (snRNA-seq) with bulk RNA sequencing (RNA-seq) enables comprehensive analysis of tumor composition while also identifying rare cellular populations and dynamic transcriptional states that may not be detectable using bulk approaches alone [18,20]. In addition, snRNA-seq allows profiling of nuclei isolated from fresh or frozen tissues, thereby reducing technical limitations associated with immediate tissue processing. These approaches have increasingly been applied to characterize tumor-associated immune populations and their functional diversity within the TME [21]. snRNA-seq was employed to comprehensively define the baseline immune landscape across ten widely used murine syngeneic tumor models, revealing model-specific differences in immune cell infiltration, composition, and transcriptional heterogeneity [22].

Although MCT2 is a high-affinity monocarboxylate transporter that mediates lactate and pyruvate transport, its role within the host tumor microenvironment (TME) remains poorly understood. In this study, we investigated the contribution of host-derived MCT2 using an inducible systemic knockout mouse model rather than targeting tumor cell-intrinsic MCT2. Given that host immune and stromal cells are continuously exposed to the lactate-rich TME, we hypothesized that MCT2 reduction would alter immune cell metabolism and TME remodeling. We focused particularly on tumor-associated macrophages (TAMs), as they are highly responsive to metabolic cues such as lactate and are key regulators of tumor progression. To define the molecular and cellular consequences of host MCT2 reduction, we compared snRNA-seq of whole tumors with bulk RNA sequencing of purified tumor-associated macrophages isolated from MCT2 knockout and control mice. This complementary transcriptomic approach enabled us to characterize changes in the tumor immune landscape and heterogeneity while identifying macrophage-specific transcriptional programs and signaling pathways through which host MCT2 reduction influences lung cancer progression.

## 2. Results

### 2.1. Tumor Biology

**Figure 1:**
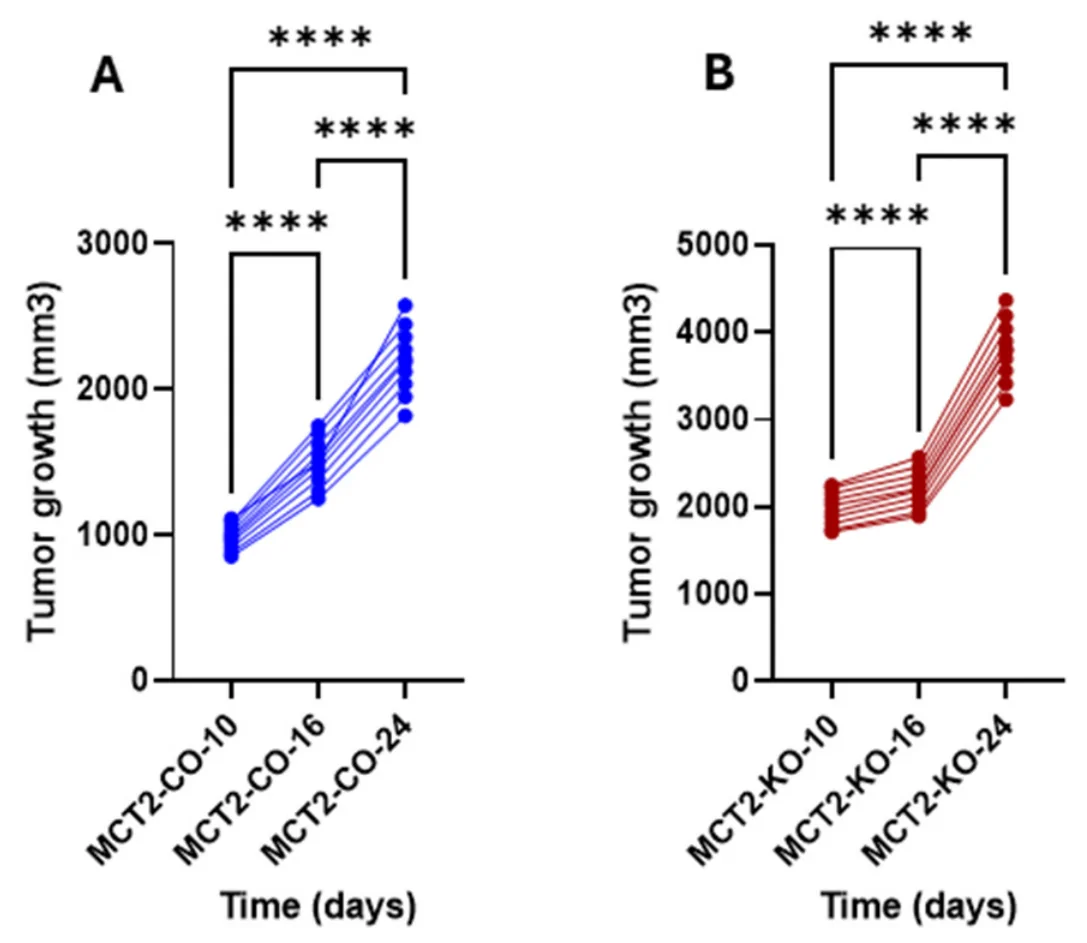
Tumor growth over time in MCT2-CO and MCT2-KO mice. (**A**) Tumor volume in CO mice measured on days 10, 16, and 24 after tumor implantation. (**B**) Tumor volume in KO mice measured at the same time points. Each line represents an individual mouse, with connected data points indicating longitudinal tumor growth. Tumor volume increased significantly over time in both CO and KO groups, with significant differences observed between days 10 and 16, days 16 and 24, and days 10 and 24 (**** *p* < 0.0001; repeated-measures one-way ANOVA with Tukey’s multiple-comparisons test). Data are presented as individual values. *n *= 10/group.

To assess differences in tumor growth between the CO and KO groups, TC1 tumor cells were injected subcutaneously into the flank and allowed to grow for 24 days. Tumors in MCT2 KO mice were significantly larger than those in MCT2 CO mice at the experimental endpoint (3882.06 ± 1313.67 mm^3^ vs. 2298.31 ± 1094.59 mm^3^, respectively; *n* = 10/group). Longitudinal measurements further demonstrated accelerated tumor growth in the MCT2 KO group throughout the study. In the MCT2 CO group, the mean tumor volume was 985 ± 86 mm^3^ on day 10, 1500 ± 160 mm^3^ on day 16, and 2200 ± 230 mm^3^ on day 24 (Figure 1A). In contrast, the MCT2 KO group exhibited mean tumor volumes of 1980 ± 194 mm^3^ on day 10, 2200 ± 240 mm^3^ on day 16, and 3800 ± 350 mm^3^ on day 24 (Figure 1B). In the CO group (A), tumor volumes were significantly greater at day 16 than at day 10 and further increased by day 24 (**** *p* < 0.0001). Similarly, in the KO group (B), tumors exhibited a marked increase in size from day 10 to day 16, followed by a further significant increase on day 24 (**** *p* < 0.0001). These findings demonstrate that host MCT2 reduction is associated with consistently accelerated tumor growth compared with the MCT2 CO group over the course of the study.

### 2.2. Single-Nucleus RNA-Seq Analysis

**Figure 2:**
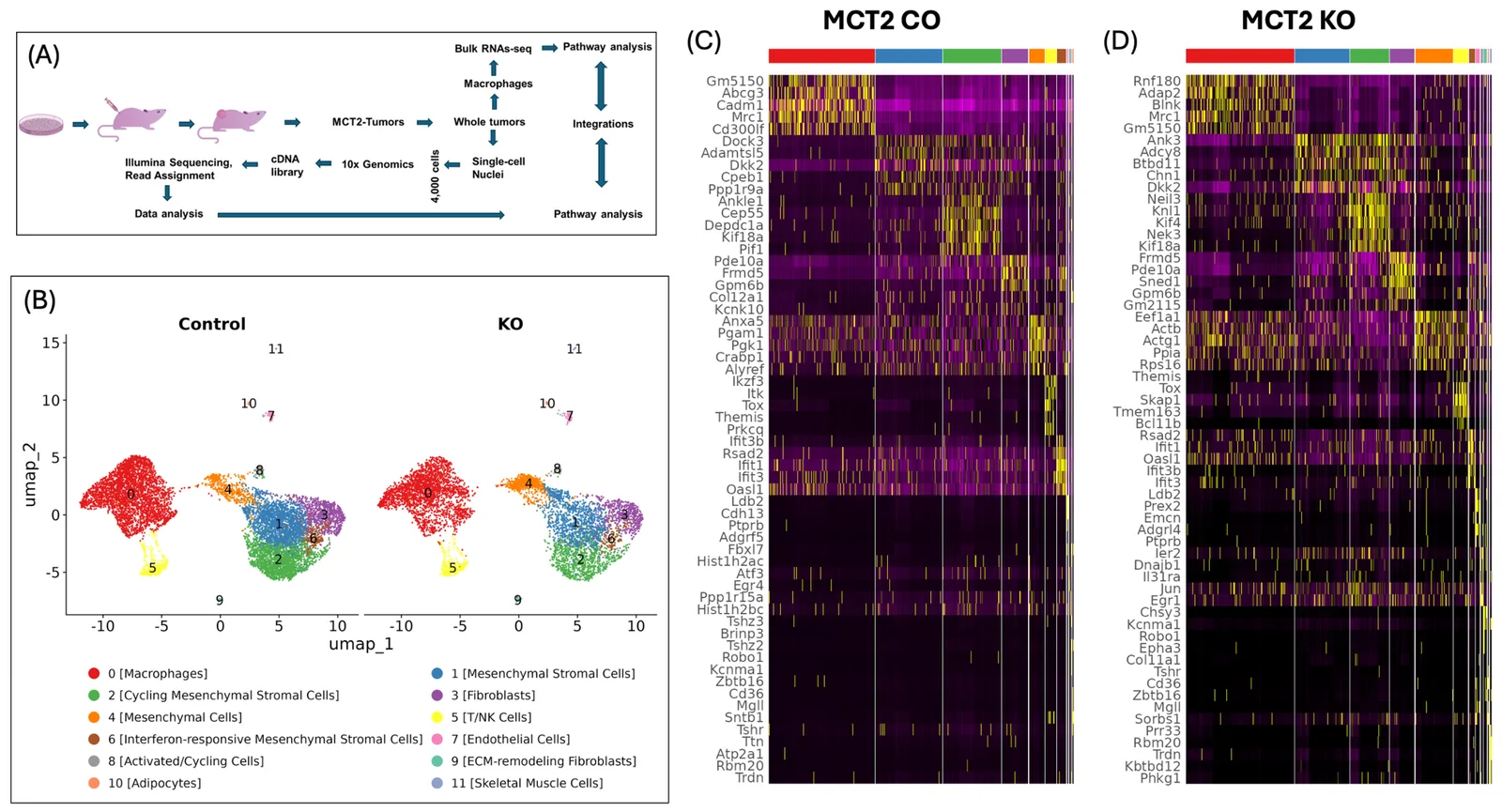
Single-nucleus transcriptomic analysis (snRNA-seq) was performed on tumors isolated from MCT2 CO and MCT2 KO mice following flank injection of TC1 cells. (**A**) Schematic illustration of the experimental design used for snRNA-seq analysis. (**B**) Uniform manifold approximation and projection (UMAP) visualization of the multi-dimensional cellular landscape in tumors from MCT2 CO and MCT2 KO mice (*n* = 3/group). Each dot represents a single nucleus. (**C**) Heatmap showing the five most enriched genes for each identified cell type in MCT2 CO. (**D**) Heatmap showing the five most enriched genes for each identified cell type in MCT2 KO. Each cell type is represented by the top five genes ranked according to the adjusted *p*-values derived from a Wilcoxon rank-sum test comparing the gene expression of each cell type with that of all other cell type populations. Each column represents the expression profile of an individual cell type. Yellow indicates the top enriched genes for each cluster. *n* = 3/group.

The experimental design shown in Figure 2A illustrates the workflow used to analyze MCT2 tumors and TAMs isolated from the same tumors. Following quality control filtering and exclusion of low-quality cells, twelve distinct cell clusters (0–11) were identified in both MCT2 CO and MCT2 KO tumors (Figure 2B). The heatmaps of the top five differentially expressed genes for each identified cell type in each condition are presented in Figure 2C,D, while the exact top five marker genes of each cluster in each condition were extracted from Tables S1 and S2 and listed in Table 1. To support Figure 2C,D as well as Table 1, dot plots in Figure S1A,B were also plotted to demonstrate high average expression and expression percentages of the top five differentially expressed genes in MCT2 CO and MCT2 KO, respectively.

Single-nucleus transcriptomic analysis identified twelve cellular clusters following TC1 cell injection, including myeloid, lymphoid, and stromal populations (Table 1). Comparison of the top five marker genes between MCT2 control (MCT2 CO) and MCT2 knockout (MCT2 KO) mice revealed differences in gene patterns in cell populations. The macrophage cluster shared *Mrc1* and *Gm5150* between genotypes but also exhibited genotype-specific markers, including *Cd300lf* and *Abcg3* in MCT2 CO and *Blnk* and *Adap2* in MCT2 KO. Likewise, the T/NK-cell cluster showed overlapping expression of *Tox* and *Themis*, whereas *Ikzf3*, *Itk*, and *Prkcq* were among the top markers in MCT2 CO and *Skap1*, *Tmem163*, and *Bcl11b* characterized the MCT2 KO cluster. Mesenchymal cells also differed in their top marker genes, with MCT2 CO characterized by *Anxa5*, *Pgam1*, *Pgk1*, *Crabp1*, and *Alyref*, whereas MCT2 KO was defined by *Eef1a1*, *Actb*, *Actg1*, *Ppia*, and *Rps16*. Adipocytes exhibited three overlaps in their top five marker genes between MCT2 CO and MCT2 KO mice, whereas endothelial and skeletal muscle cells retained two shared markers despite differences in the remaining top-ranked genes.

**Figure 3:**
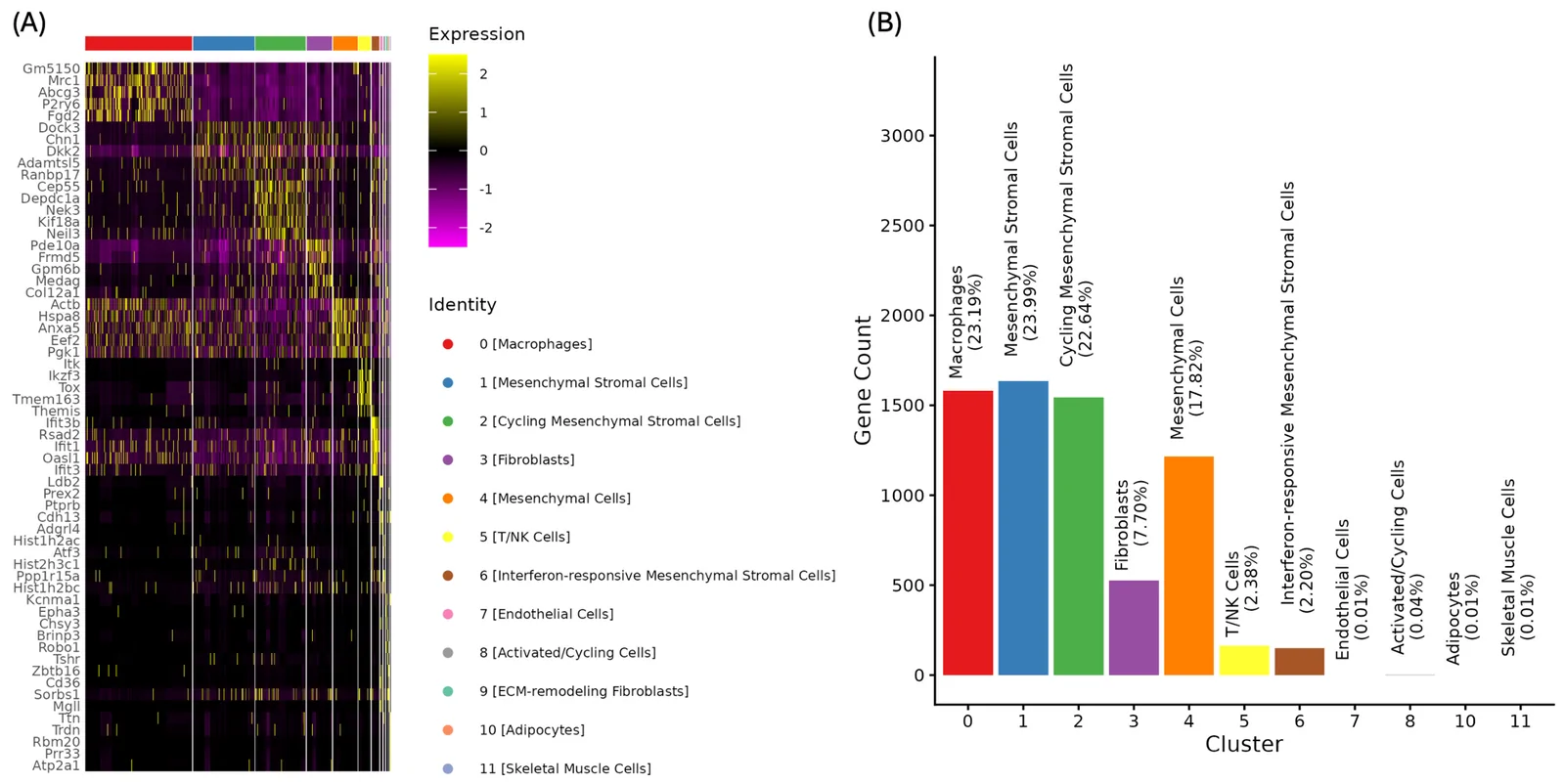
snRNA-seq analysis of statistically significant differentially expressed genes (DEGs) in MCT2 KO and MCT2 CO mice injected in the flank with lung cancer cells for 24 days. (**A**) Heatmap showing the top five differentially expressed genes (DEGs) for each of the 12 cell clusters (clusters 0–11). Marker genes were identified using the Seurat FindAllMarkers function and ranked by adjusted *p*-values from a Wilcoxon rank-sum test. Rows represent genes, and columns represent individual nuclei grouped by clusters. Heatmap colors indicate scaled gene expression, with higher expression shown in yellow and lower expression in purple. Three biological replicates were included for each condition (*n* = 3/group). (**B**) Bar plot showing the numbers and percentages of significant differentially expressed genes (DEGs) identified in each cell cluster following comparison of MCT2 KO over MCT2 CO samples. Differential expression analysis was performed separately for each cell cluster using the Seurat FindMarkers function. Genes with an adjusted *p*-value ≤ 0.05 and an absolute log2 fold change ≥ 0.58 were considered significantly differentially expressed. The numbers and percentages of significant DEGs in each cluster are summarized in the bar plot. *n* = 3/group.

Analysis of differentially expressed marker genes using the Seurat FindAllMarkers function with integration of MCT2 KO and MCT2 CO mice identified distinct transcriptional signatures across all cell populations (Figure 3A and Table 2). Figure 3A and Table 2, as well as the dot plot in Figure S2, summarize high expression of the top five differentially expressed marker genes, extracted from the complete set in Table S3, for each cell population. A heatmap for the top five DEGs of each cluster in integrated MCT2 KO and MCT2 CO is shown in Figure 3A. Macrophages were characterized by enrichment of *Gm5150*, *Mrc1*, *Abcg3*, *P2ry6*, *Fgd2*, while T/NK cells showed increased expression of *Itk*, *Ikzf3*, *Tox*, *Tmem163*, and *Themis*.

For the MCT2 knockout (KO) versus control (CO) differential expression analysis, the numbers and proportions of differentially expressed genes (DEGs) identified by the Seurat FindMarkers function for each cell type are shown in Figure 3B. Macrophages, mesenchymal stromal cells, and cycling mesenchymal stromal cells roughly exhibited similar proportions (~22–24%) of DEGs. The top five DEGs for each cell type (Table 3; extracted from Table S4) revealed both conserved and cell-type-specific transcriptional alterations associated with MCT2 deficiency. Macrophages exhibited differential expression of genes including *Bnc2*, *Gulp1*, *Itgb8*, *Nox4*, and *Zfpm2*, suggesting alterations in extracellular matrix interactions, oxidative signaling, and phagocytic function. Mesenchymal stromal cell populations, including cycling and interferon-responsive subsets, showed widespread transcriptional changes characterized by differential expression of *Arhgap15*, *Dock2*, *Elmo1*, *Lhfpl3*, *Nxph1*, *Rnf150*, *Unc5c*, and *Itgb8*, indicating remodeling of cell migration, signaling, and stromal organization. Fibroblasts displayed altered expressions of *Adam12*, *Dkk2*, *Ebf2*, *Prkg1*, and *Pstpip2*, consistent with changes in extracellular matrix remodeling and stromal regulation. T/NK cells exhibited differential expressions of *Adgrl3*, *Ghr*, *Mecom*, *Pbx1*, *Rbms3*, and *Sema7a*, suggesting altered immune cell signaling and differentiation. Collectively, these findings indicate that MCT2 deletion induces cell-type-specific transcriptional reprogramming, with the most extensive transcriptional changes occurring in mesenchymal stromal cell populations and macrophages.

#### 2.2.1. Functional Enrichment Analysis of Single-Nucleus RNA-Seq

Next, we identified genes involved in several key metabolic pathways, including glycolysis, tricarboxylic acid (TCA) cycle, oxidative phosphorylation (OXPHOS), and fatty acid metabolism. These pathways are particularly important in lung cancer because tumor cells undergo extensive metabolic reprogramming to support their growth and survival. Rather than relying on normal cellular metabolism, cancer cells often rewire metabolic processes to generate energy more efficiently, produce biosynthetic precursors, adapt to cellular stress, evade therapeutic interventions, and promote metastasis [23,24]. A hallmark of this reprogramming is enhanced aerobic glycolysis (the Warburg effect), which allows cancer cells to sustain rapid proliferation [25]. In addition, tumor cells can bypass conventional mitochondrial glucose utilization and instead fuel the TCA cycle through alternative carbon sources, enabling the maintenance of oxidative phosphorylation and metabolic flexibility under diverse environmental conditions [26].

**Figure 4:**
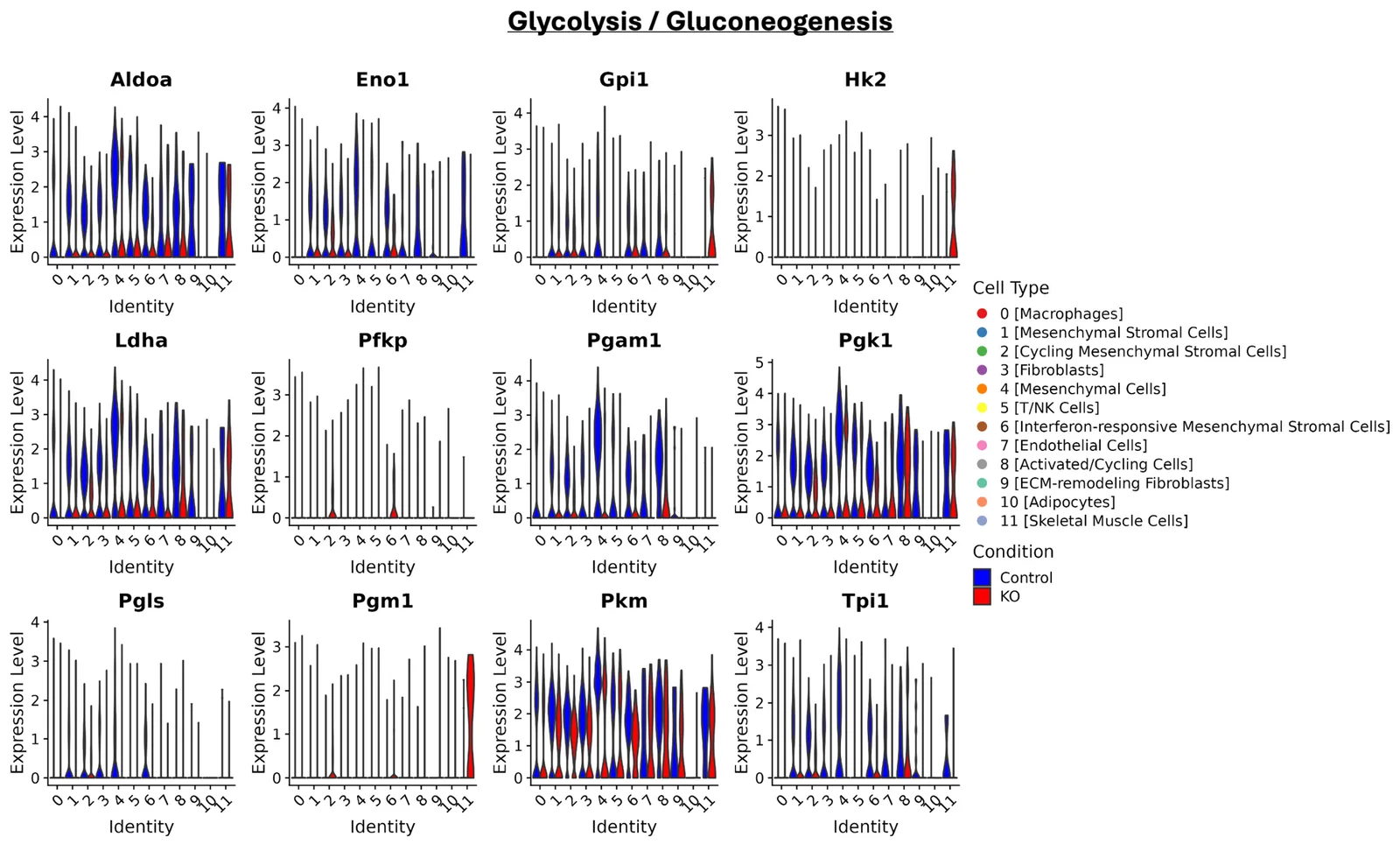
Glycolysis gene expressions in MCT2 KO and MCT2 CO. Violin plots show expression of selected glycolysis pathway genes across cell types in snRNA-seq data. Genes were selected based on differential expressions between MCT2 KO (red) and MCT2 CO (blue) samples. The x-axis indicates clusters (cell types); the y-axis shows normalized expression levels.

In glycolysis, we examined the expression of glycolysis-related genes across KO and CO cell populations (Figure 4; Tables S5–S7). Violin plots illustrate the single-nucleus expression of 12 glycolysis-related genes across the major cell clusters (Figure 4). Among these genes, the core glycolytic enzymes *Aldoa*, *Eno1*, *Ldha*, *Pgam1*, *Pgk1*, and *Pkm* were broadly expressed across multiple cell populations. Differential expression analysis revealed coordinated downregulation of glycolytic genes in KO cells. In macrophages, *Ldha*, *Eno1*, *Hk2*, *Pgam1*, *Pgk1*, *Pgls*, and *Tpi1* were significantly downregulated in KO compared with Control. Collectively, these findings demonstrate that MCT2 deficiency is associated with broad suppression of the glycolytic transcriptional program across multiple stromal and immune cell populations.

**Figure 5:**
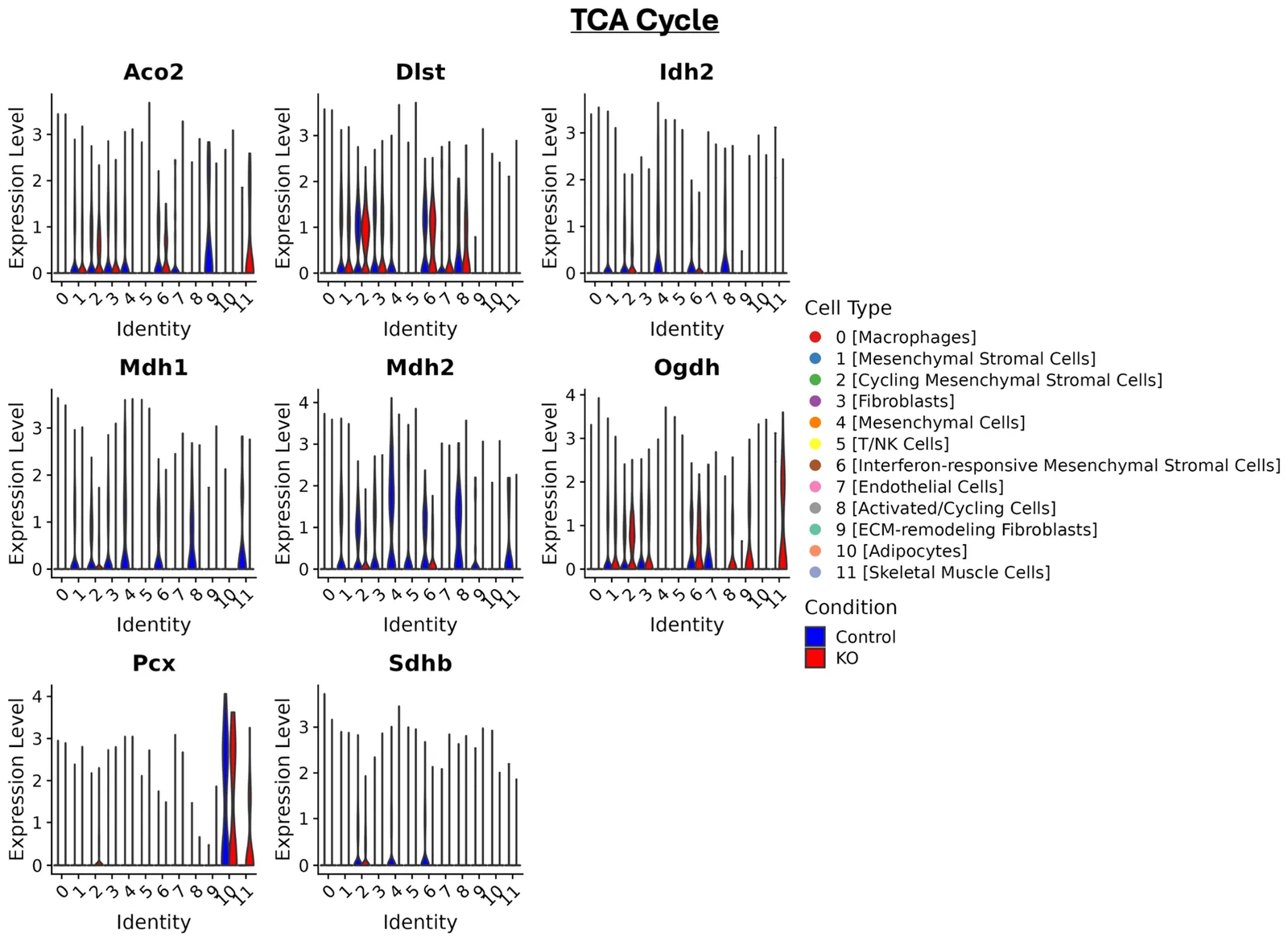
TCA Cycle (Citric Acid Cycle/Krebs Cycle) Pathway gene expression and network changes in the KO tumors. Violin plots for TCA selected genes and their expression in snRNA-seq in each cell type, using differentially expressed genes between the knockouts (KOs) and controls (COs) in the expression profiles. The x-axis indicates cluster (cell type); the y-axis shows normalized expression levels.

In the TCA cycle pathway, eight TCA-cycle-related genes (*Aco2*, *Dlst*, *Idh2*, *Mdh1*, *Mdh2*, *Ogdh*, *Pcx*, and *Sdhb*) were examined across KO and CO cell populations (Figure 5; Tables S8–S10). Among these genes, *Mdh1* and *Mdh2* were significantly downregulated in KO macrophages compared with CO, indicating reduced expression of key malate dehydrogenases involved in the TCA cycle. Similar reductions in *Mdh1* and *Mdh2* were also observed in several mesenchymal cell populations, whereas *Aco2*, *Idh2*, and *Sdhb* were downregulated and *Pcx* was upregulated only in selected mesenchymal populations. Overall, these findings indicate that suppression of *Mdh1* and *Mdh2* is a prominent transcriptional feature of MCT2-reduction macrophages and is accompanied by broader alterations in TCA-cycle-related gene expression across other stromal cell populations.

**Figure 6:**
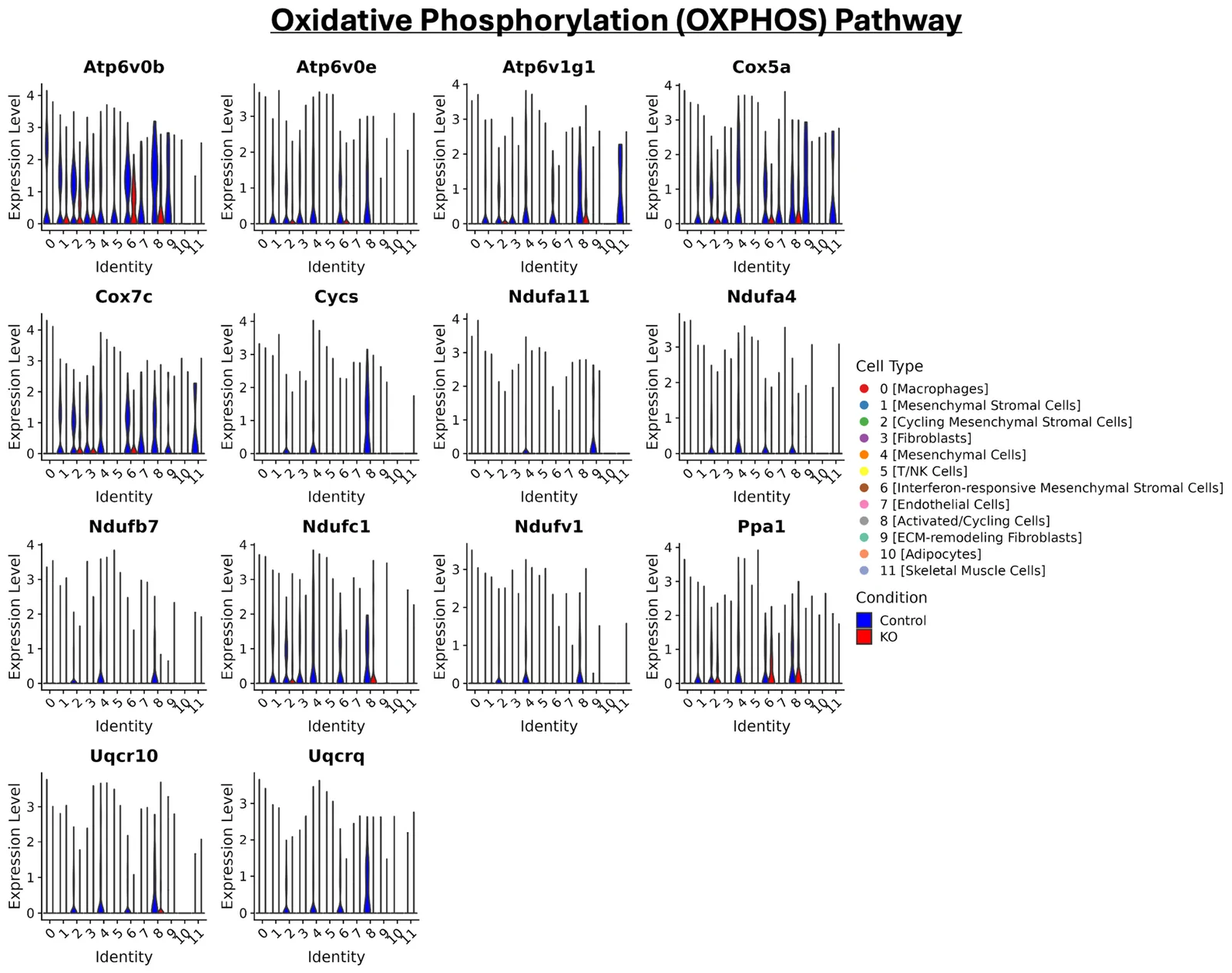
Oxidative phosphorylation (OXPHOS) pathway gene expression in MCT2 KO and MCT2 CO. Violin plots showing expression of selected OXPHOS-related genes across cell types in snRNA-seq data. Genes were identified based on differential expression between MCT2 KO (red) and MCT2 CO (blue) samples. The x-axis indicates cell type clusters; the y-axis shows normalized expression levels.

Analysis of oxidative phosphorylation (OXPHOS)-related genes revealed the coordinated downregulation of multiple nuclear-encoded genes in KO compared with control cells across several cell populations (Figure 6, Tables S11–S13). Analyses consistently identified reduced expression of *Atp6v0b*, *Atp6v0e*, *Atp6v1g1*, *Cox5a*, *Cox7c*, *Cycs*, *Ndufa4*, *Ndufa11*, *Ndufb7*, *Ndufc1*, *Ndufv1*, *Ppa1*, *Uqcr10*, and *Uqcrq*. This coordinated reduction was observed in multiple cell populations. Notably, the downregulated genes include components of multiple electron transport chain complexes, including Complex I (*Ndufa11*, *Ndufb7*, *Ndufc1*, and *Ndufv1*), Complex III (*Uqcr10* and *Uqcrq*), Complex IV (*Cox5a* and *Cox7c*), and the electron carrier *Cycs*, indicating coordinated transcriptional suppression of genes involved in the oxidative phosphorylation pathway. Hence, these findings demonstrate coordinated suppression of electron transport chain and energy metabolism-associated gene expression in MCT2-reduction macrophages, consistent with reduced oxidative phosphorylation-related transcription.

**Figure 7:**
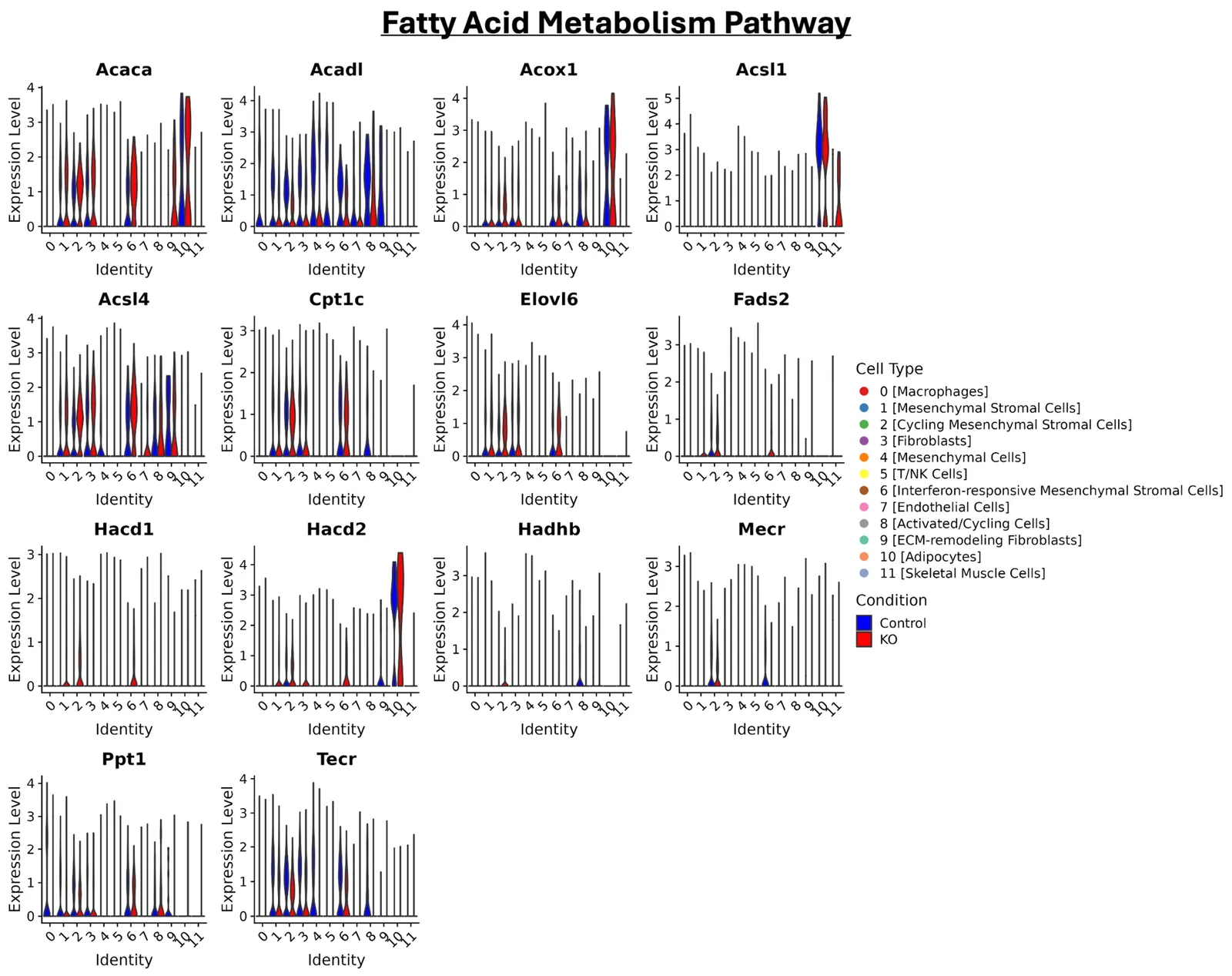
Fatty acid metabolism pathway gene expression in MCT2 KO and MCT2 CO. Violin plots show the expression of selected fatty acid metabolism-related genes across cell types in snRNA-seq data. Genes were identified based on differential expression between MCT2 KO (red) and MCT2 CO (blue) expression profiles. The x-axis indicates cell type clusters; the y-axis shows normalized expression levels.

Differential expression analysis of fatty acid metabolism-related genes revealed distinct transcriptional remodeling in response to MCT2 deficiency (Figure 7; Tables S14–S16). In macrophages, *Acadl*, a key enzyme involved in fatty acid β-oxidation, was significantly downregulated, whereas *Acsl4*, *Acaca*, and *Cpt1c* were significantly upregulated in KO compared with Control. These findings indicate coordinated transcriptional changes affecting fatty acid activation, synthesis, and mitochondrial fatty acid transport in macrophages. Similar patterns were observed in mesenchymal stromal populations, where *Acadl* remained consistently downregulated and *Acsl4* and *Elovl6* were upregulated, while the fatty acid elongation-associated genes *Mecr* and *Tecr* were significantly downregulated. Therefore, these results demonstrate that MCT2 deficiency is associated with coordinated remodeling of fatty acid metabolism-related gene expression, characterized by reduced expression of β-oxidation-associated genes together with increased expression of genes involved in fatty acid activation and lipid biosynthesis, with macrophages exhibiting a prominent metabolic remodeling signature.

**Figure 8:**
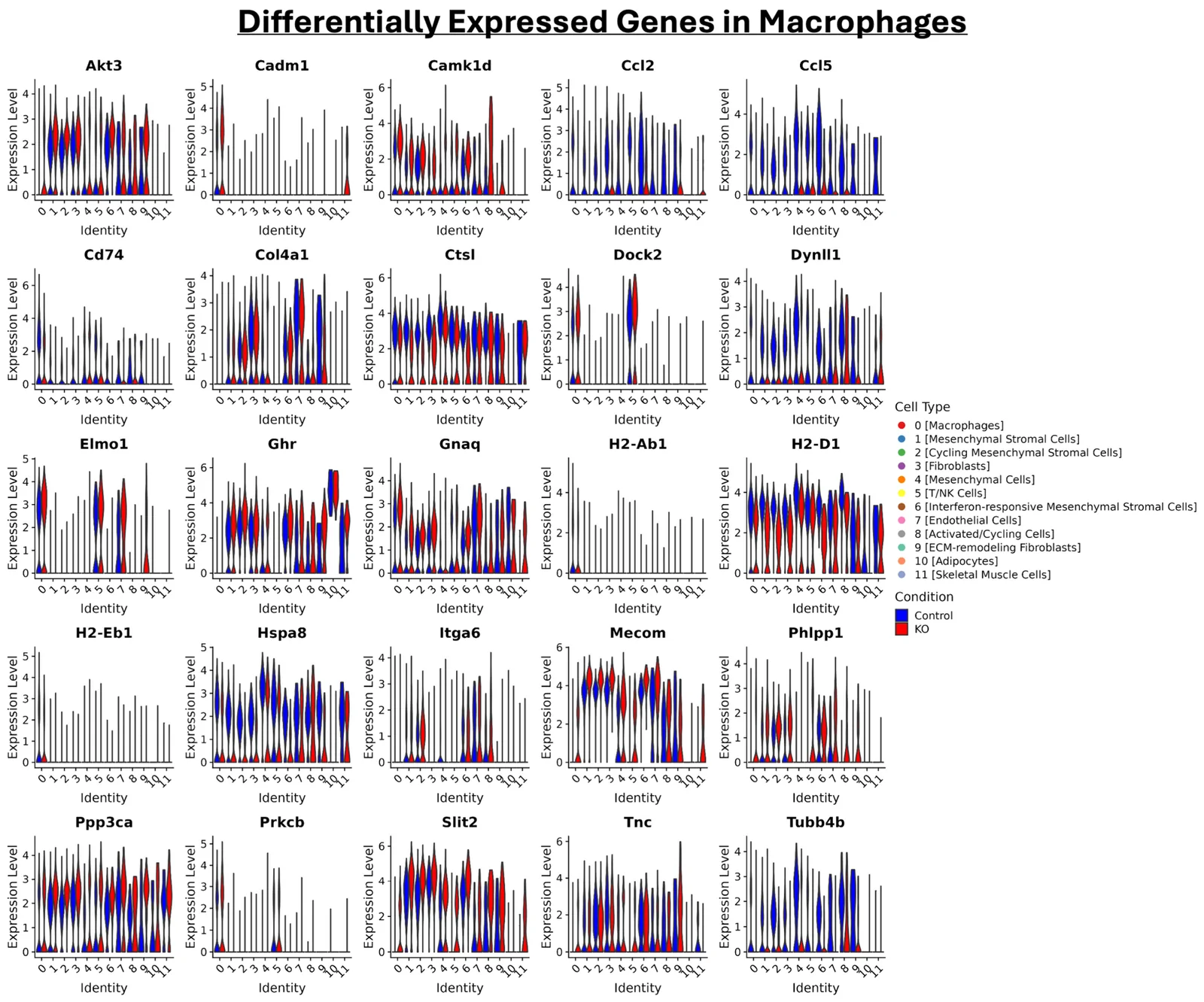
Differentially expressed genes in macrophages from Control (CO) and Knockout (KO) samples. Violin plots show the expression of selected differentially expressed genes (DEGs) in macrophage clusters from Control (blue) and KO (red) samples. The violin width reflects the distribution (density) of cells at each normalized expression level for a given gene within each cell type. The x-axis represents cell types, and the y-axis represents normalized gene expression levels.

Next, we identified differentially expressed genes (DEGs) and associated KEGG pathways in MCT2 KO macrophages compared with MCT2 CO macrophages (Figure 8; Table 4; Tables S17–S19). KO macrophages showed marked transcriptional remodeling, with strong upregulation of *Mecom* and *Slit2*, together with genes involved in intracellular signaling, cell adhesion, and extracellular matrix interactions, including *Akt3*, *Cadm1*, *Col4a1*, *Dock2*, *Elmo1*, *Gnaq*, *Itga6*, *Prkcb*, and *Tnc*. In contrast, genes associated with antigen processing and presentation, including *Cd74*, *Ctsl*, *H2-Ab1*, *H2-D1*, and *H2-Eb1*, were significantly downregulated, as was the chemokine *Ccl5*. KEGG pathway analysis linked these DEGs to chemokine, PI3K-Akt, MAPK, antigen-presentation, cell-adhesion, focal-adhesion, ECM-receptor, and cytokine-signaling pathways. Collectively, these findings indicate that MCT2 deficiency reshapes macrophage transcriptional programs by increasing signaling, adhesion, and extracellular matrix-associated gene expression while suppressing antigen processing and presentation.

#### 2.2.2. Cell Trajectory and Lineage Analysis

**Figure 9:**
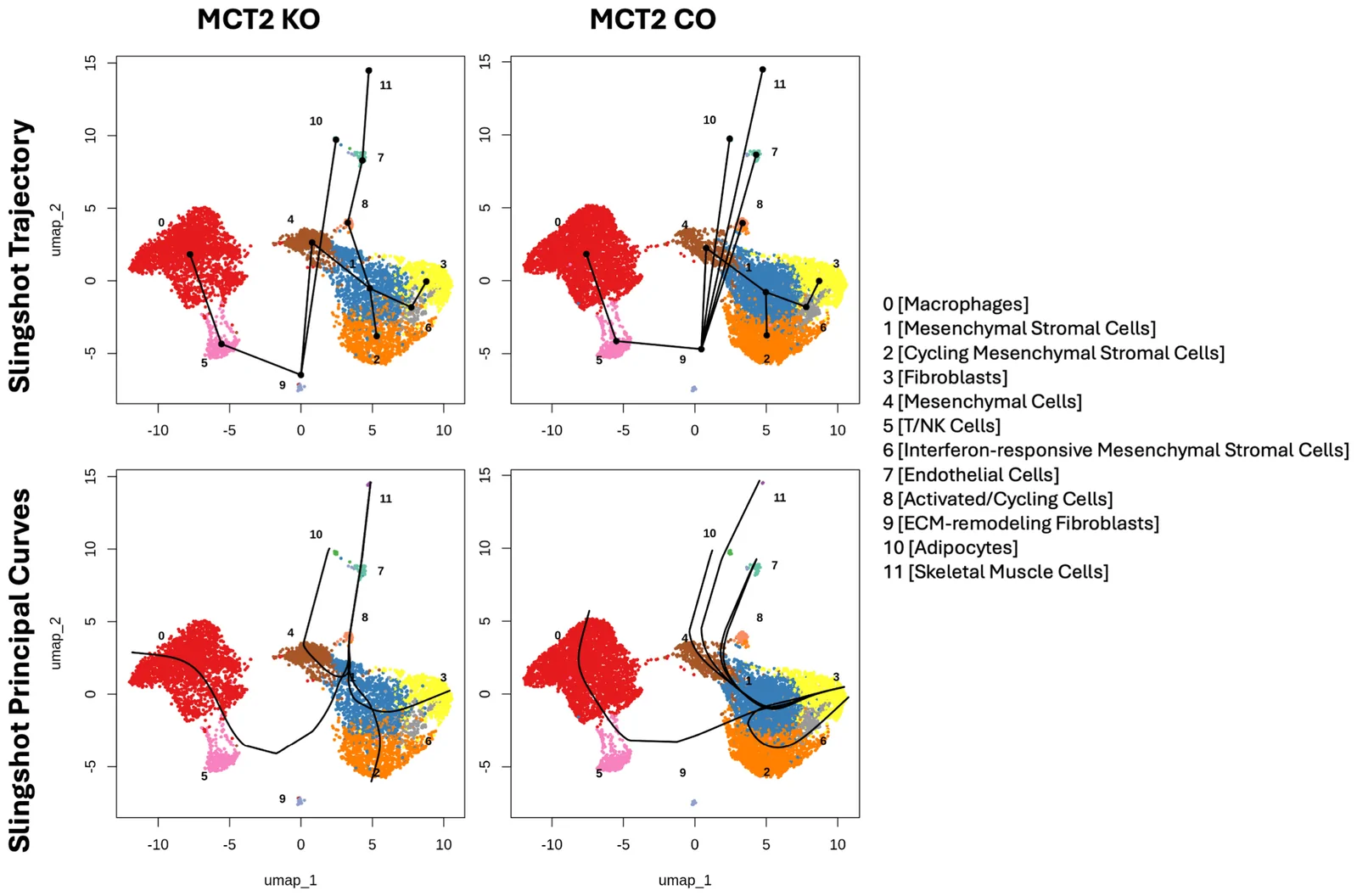
Slingshot trajectory analysis of MCT2 knockout (KO) and control (CO) single-nucleus RNA-seq (snRNA-seq) populations. Cells are displayed in UMAP space and colored by annotated cell type. The top panels show the Slingshot minimum spanning trees, in which black nodes indicate cluster centers and connecting lines represent the inferred relationships among cell populations. The bottom panels show the fitted principal curves representing putative lineages and the corresponding pseudotime trajectories. Although the overall lineage architecture was largely conserved between MCT2 KO and CO, differences in trajectory topology and principal-curve geometry were observed, suggesting altered cell-state organization and lineage progression following MCT2 reduction.

Using single-nucleus RNA-seq data, we applied Slingshot trajectory analysis to infer putative lineage relationships among cell populations in MCT2 control (CO) and MCT2 knockout (KO) samples (Figure 9). Comparing the inferred trajectories, the most notable differences were observed in the trajectory topology involving endothelial cells (cluster 7), activated/cycling cells (cluster 8), and skeletal muscle cells (cluster 11). Further examinations of the principal curves (Figure 9) and the inferred pseudotime (Figure S3) showed that Slingshot positioned the putative lineage origin near the skeletal muscle cells (cluster 11) in KO samples and near the fibroblasts (cluster 3) in CO samples. Differences in the shapes and positions of the fitted principal curves further suggest that MCT2 deficiency may alter cell-state organization and transitions along the inferred trajectories. However, these trajectories represent computationally inferred lineage relationships and should not be interpreted as direct evidence of lineage progression.

#### 2.2.3. Cell–Cell Interaction Analysis

**Figure 10:**
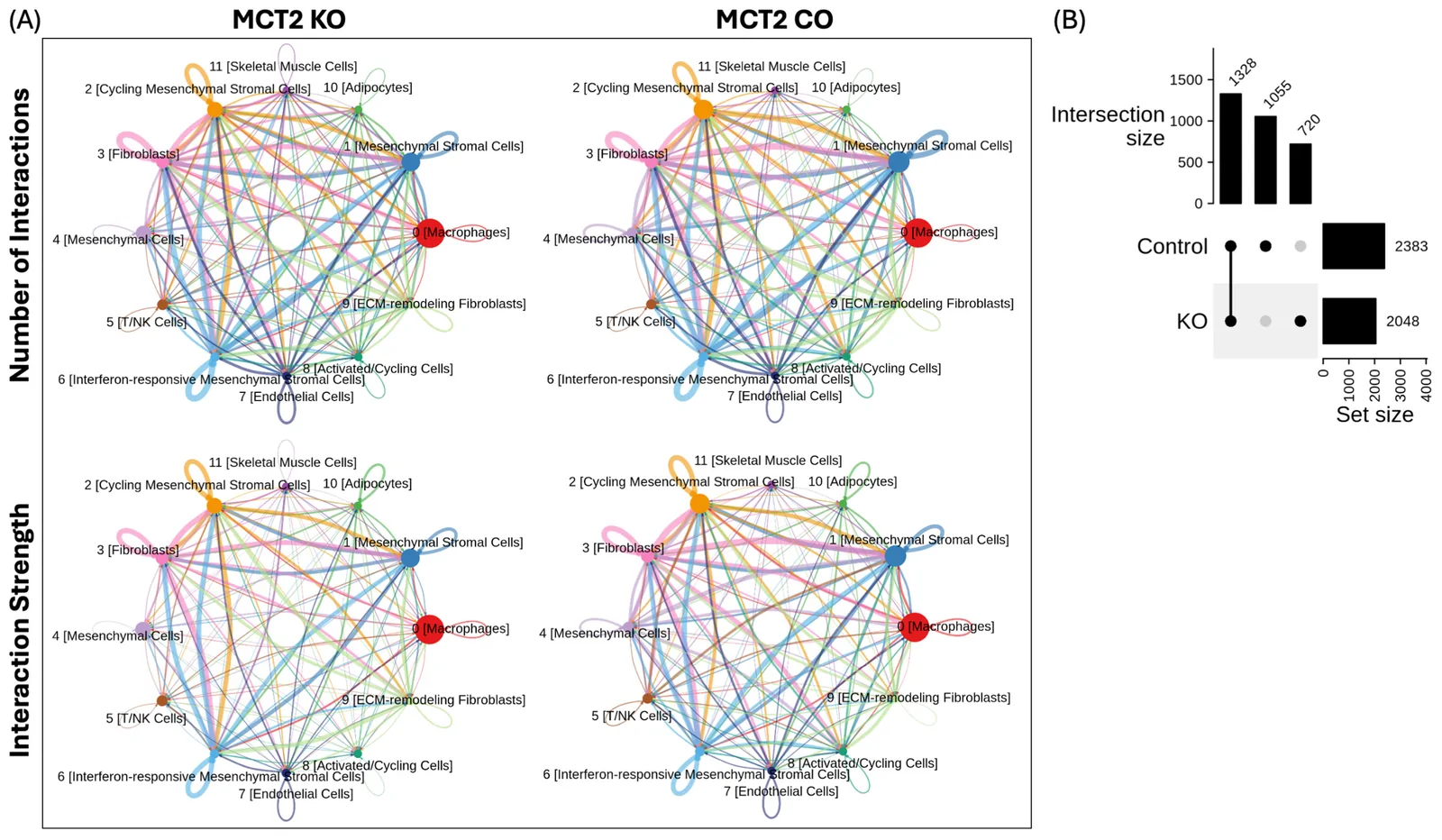
CellChat analysis of cell–cell communication networks in MCT2 knockout (KO) and MCT2 control (CO) samples. (**A**) Global cell–cell communication networks inferred by CellChat for the MCT2 KO and CO groups. The upper panels show the number of inferred ligand–receptor interactions, whereas the lower panels depict the overall interaction strength (communication probability) between cell populations. Each node represents a cell type, with node size proportional to the total number of interactions involving that cell population. Edge thickness corresponds to the number of interactions (upper panels) or the cumulative interaction strength (lower panels), and edge colors indicate the signaling source (sender) cell type. (**B**) UpSet plot summarizing the overlap and uniqueness of inferred ligand–receptor interactions between the MCT2 CO and KO groups. Black bars indicate the number of shared and condition-specific interactions, whereas horizontal bars represent the total number of inferred interactions in each group.

In the cell–cell interaction analysis, the communication networks shown in Figure 10A revealed broadly similar intercellular communication architectures between MCT2 knockout (KO) and control (CO) conditions. However, the UpSet plot in Figure 10B showed that the MCT2 CO condition contained a greater number of predicted ligand–receptor interactions than the MCT2 KO condition, with 1,328 common interactions existing in both CO and KO, 1055 distinct interactions existing only in CO, and 720 distinct interactions existing only in KO. Apart from CellChat analyses for KO and CO conditions, CellChat analyses for 6 samples (3 knockout samples and 3 control samples) were also executed independently. The figure with the number of interactions of independent samples is presented in Figure S4, whereas the figure with interaction strength of independent samples is displayed in Figure S5. The complete ligand–receptor interaction data are presented in Table S20. In six samples independent analyses, the KO group showed a lower average number of ligand–receptor interactions than the CO group. These findings suggest that MCT2 deficiency is associated with an overall reduction in predicted cell–cell communication. Consistent with this observation, the control network exhibited slightly stronger and denser intercellular communication.

Biologically, these findings suggest that MCT2 deficiency may alter the coordination of metabolic and inflammatory signaling within the tissue microenvironment. As MCT2 mediates the transport of monocarboxylates, including lactate, its reductions may disrupt lactate exchange between metabolically active cells and neighboring stromal, immune, adipocyte, endothelial, and skeletal muscle cell populations. The reduced number of predicted ligand–receptor interactions in the MCT2 knockout (KO) condition suggests a less complex intercellular communication network, potentially reflecting impaired crosstalk involved in tissue remodeling, immune regulation, angiogenesis, and metabolic adaptation. Although MCT2 deletion does not appear to abolish cell–cell communication, it may reduce the diversity of intercellular signaling and shift the tissue microenvironment toward weaker or more selective stromal-immune interactions, thereby limiting adaptive tissue remodeling.

### 2.3. Flow Cytometry for Macrophage (CD11b)

**Figure 11:**
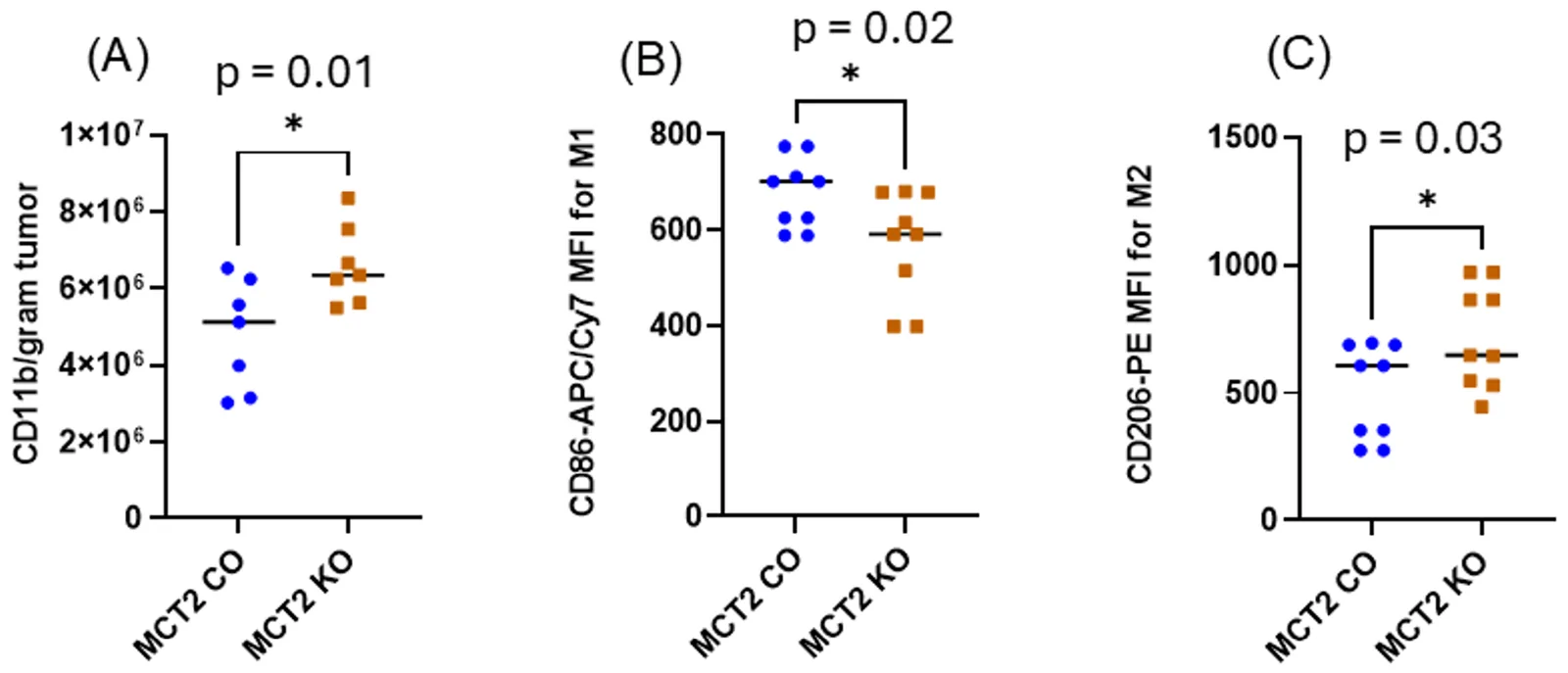
Flow cytometric analysis of tumor-infiltrating CD11b^+^ cells and their polarization in the KO and CO mice. (**A**) CD11b^+^ cell counts per gram of tumor tissue were higher in KO mice compared to controls. (**B**) Mean fluorescence intensity (MFI) of CD86 (M1 marker) was reduced in CD11b^+^ cells from the KO mice. (**C**) MFI of CD206 (M2 marker) was elevated in CD11b^+^ cells from the KO tumors, indicating enhanced M2 polarization. An unpaired two-tailed t-test was used. *n* = 7–8/group. * indicates statistically significant *p* < 0.05.

Next, we isolated and characterized CD11b^+^ cells from tumors and measured the expression of polarization markers CD86 (M1) and CD206 (M2) in both groups of mice (Figure 11). The number of CD11b^+^ cells per gram of tumor tissue was significantly higher in KO mice compared to CO mice, as shown in Figure 11A. Flow cytometric analysis revealed that the mean fluorescence intensity (MFI) of the M1 marker CD86 was significantly reduced in the KO group (*p* = 0.02) (Figure 11B). In contrast, the MFI for the M2 marker CD206 was elevated in KO tumors compared to CO (*p* = 0.03) (Figure 11C). The observed increase in CD11b^+^ abundance and shift toward an M2-like polarization state in the KO condition provides biologically meaningful insight, suggesting that the reduction of MCT2 promotes an immunosuppressive tumor microenvironment.

### 2.4. Bulk RNA-Seq Analysis

**Figure 12:**
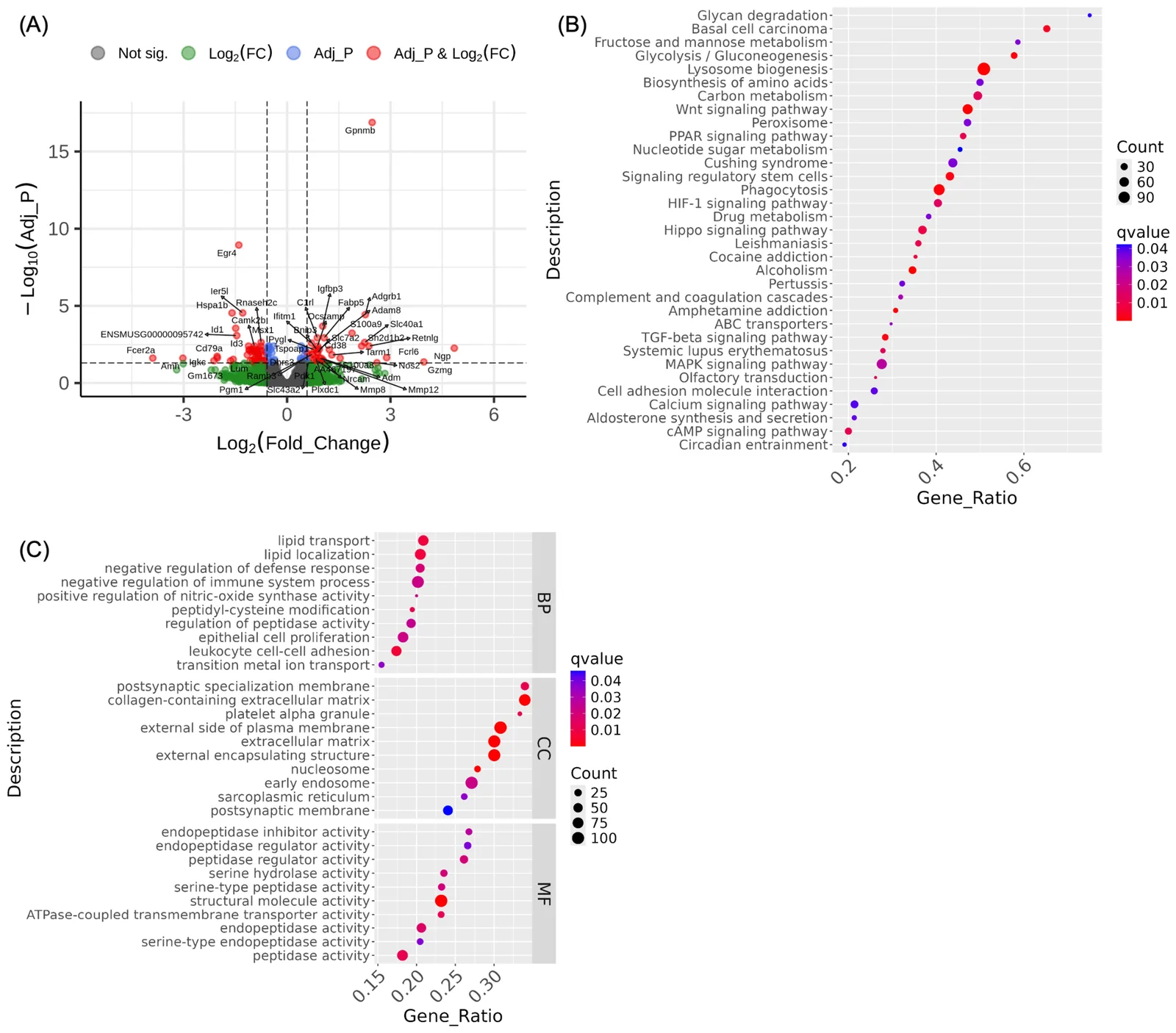
Differential expression and functional enrichment analyses of bulk RNA-seq data. (**A**) Volcano plot showing differentially expressed genes between the two groups. The red dots represent significantly upregulated genes (log_2_ fold change ≥ 0.58 and −log_10_ adjusted *p*-value ≥ 1.3) and downregulated genes (log_2_ fold change ≤ 0.58 and −log_10_ adjusted *p*-value ≥ 1.3). The green dots are insignificant genes below defined −log_10_ adjusted *p*-value threshold. The blue dots are insignificant genes below defined absolute log_2_ fold change threshold. The gray dots are insignificant genes below both defined thresholds. Selected significantly altered genes are labeled. (**B**) KEGG pathway enrichment analysis of differentially expressed genes. The top enriched pathways are displayed according to gene count and statistical significance. (**C**) Gene Ontology (GO) enrichment analysis of differentially expressed genes, including Biological Process (BP), Cellular Component (CC), and Molecular Function (MF) categories. Bubble size indicates gene count, and color represents enrichment significance. *n* = 3/group.

Since macrophages play an important role in tumors, we purified CD11b^+^ from both MCT2 CO and MCT2 KO and performed bulk RNA-seq. The bulk RNA-seq differential expression analysis revealed 34 upregulated genes and 46 downregulated genes. The differential expression analysis identified numerous significantly dysregulated genes between groups (Figure 12A). Notably, *Gpnmb, Adgrb1*, *Adam8*, and *Fcrl6* were among the most up-regulated genes, whereas *Egr4*, *Ier5l*, and *Hspa1b* were significantly down-regulated.

#### Functional Enrichment Analysis of Bulk RNA-Seq

KEGG pathway analysis revealed significant enrichment in pathways related to metabolism, lysosomal function, immune responses, and signaling, including glycolysis/gluconeogenesis, lysosome, phagosome, Wnt, PPAR, HIF-1, and MAPK signaling pathways (Figure 12B).

GO enrichment analysis showed that the differentially expressed genes were mainly associated with lipid transport, immune regulation, and epithelial cell proliferation in the Biological Process category. Enriched Cellular Component terms included the extracellular matrix and plasma membrane, while Molecular Function terms were dominated by endopeptidase inhibitor/regulator activity and transporter activity (Figure 12C).

### 2.5. Gene Network Analysis

**Figure 13:**
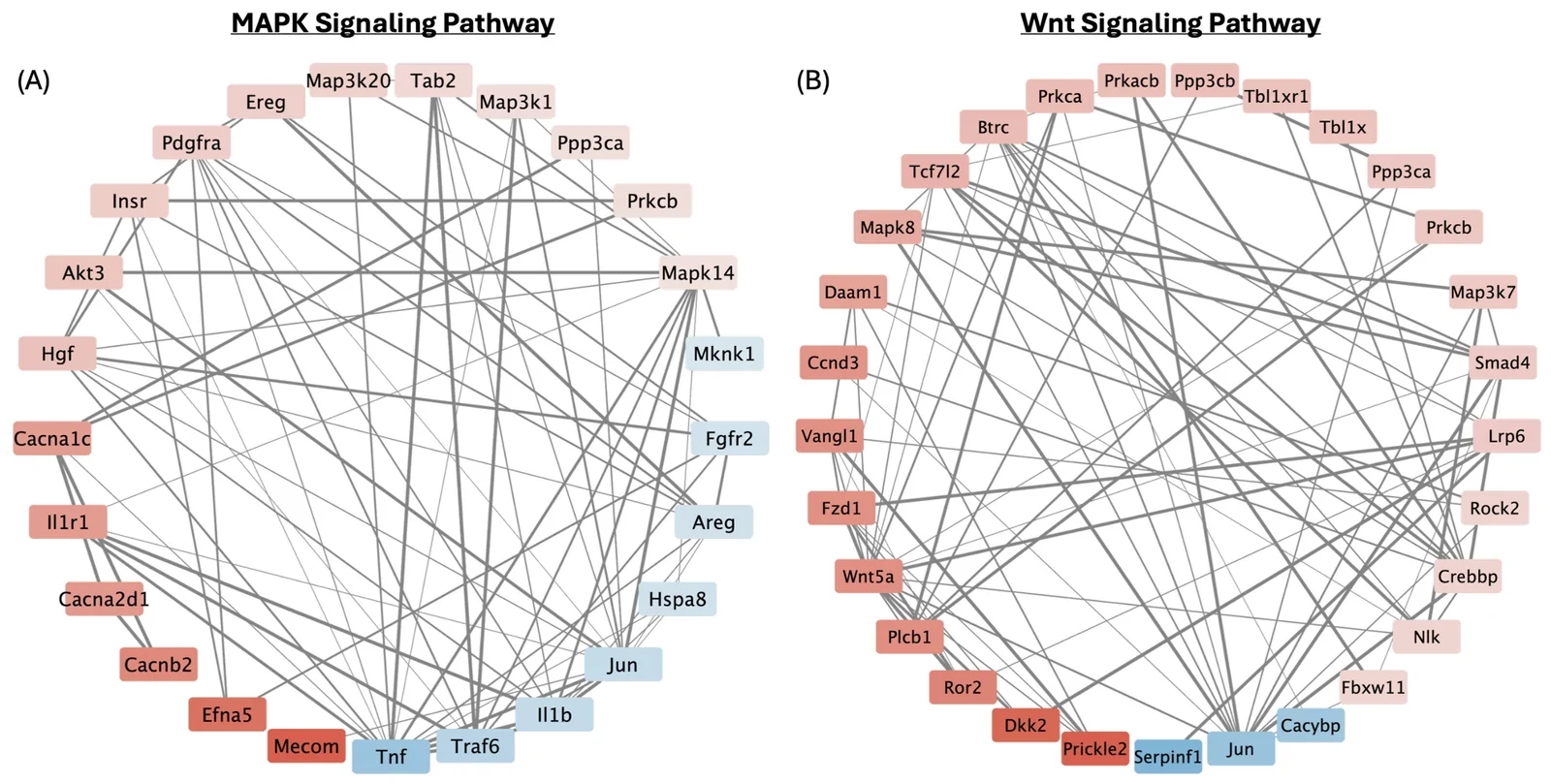
Shared MAPK and Wnt signaling pathways identified from macrophage snRNA-seq and bulk RNA-seq analyses. (**A**) Protein–protein interaction (PPI) network of representative genes associated with the MAPK signaling pathway. (**B**) PPI network of representative genes associated with the Wnt signaling pathway. Node colors represent relative gene expression changes between MCT2 knockout (KO) and control (CO) macrophages, with red indicating upregulated genes and blue indicating downregulated genes. Gene expression changes are shown as log_2_ fold changes derived from Seurat FindMarkers analysis comparing KO and CO macrophage clusters.

To further investigate the biological significance of the identified gene networks, significantly enriched pathways from macrophage snRNA-seq and bulk RNA-seq analyses were compared. The MAPK and Wnt signaling pathways emerged as one of the most significantly enriched pathways shared between the two datasets (Figure 13). The top 30 differentially expressed genes from each pathway were then selected for protein–protein interaction analysis using the STRING database [27], and the resulting networks were visualized and analyzed in Cytoscape v3.10.2 [28].

Protein–protein interaction network analysis of representative MAPK pathway genes revealed coordinated changes involving growth factor signaling (*Pdgfra*, *Fgfr2*, *Hgf*, and *Areg*), MAPK cascade components (*Map3k1*, *Map3k20*, *Mapk14*, and *Mknk1*), inflammatory mediators (*Tnf*, *Il1b*, *Il1r1*, and *Traf6*), calcium signaling (*Cacna1c*, *Cacna2d1*, and *Cacnb2*), and stress-responsive transcription (*Jun*). Several genes, including *Mecom*, *Efna5*, and *Tnf*, were upregulated, whereas others, such as *Fgfr2*, *Jun*, *Il1b*, and *Mknk1*, were downregulated, indicating selective remodeling of MAPK signaling rather than uniform pathway activation or suppression.

The Wnt signaling pathway also exhibited coordinated alterations in genes involved in ligand–receptor interactions (*Wnt5a*, *Fzd1*, *Ror2*, and *Lrp6*), non-canonical Wnt signaling (*Vangl1*, *Plcb1*, and *Daam1*), intracellular signaling mediators (*Prkca*, *Prkcb*, *Map3k7*, and *Rock2*), transcriptional regulation (*Tcf7l2*, *Tbl1x*, and *Tbl1xr1*), and downstream effectors (*Jun*, *Cacybp*, and *Serpinf1*). Differential expressions of genes such as *Wnt5a*, *Fzd1*, *Ror2*, *Dkk2*, and *Prickle2*, together with reduced expression of *Jun*, *Cacybp*, and *Serpinf1*, suggest substantial rewiring of Wnt signaling networks in MCT2-reduction macrophages.

Collectively, these findings indicate that MCT2 deficiency remodels interconnected MAPK- and Wnt-mediated signaling pathways to potentially alter inflammatory responses, growth factor signaling, and macrophage communication within the tumor microenvironment.

### 2.6. Comparisons of snRNA-Seq, Pseudobulk, and Bulk RNA-Seq Analyses

**Figure 14:**
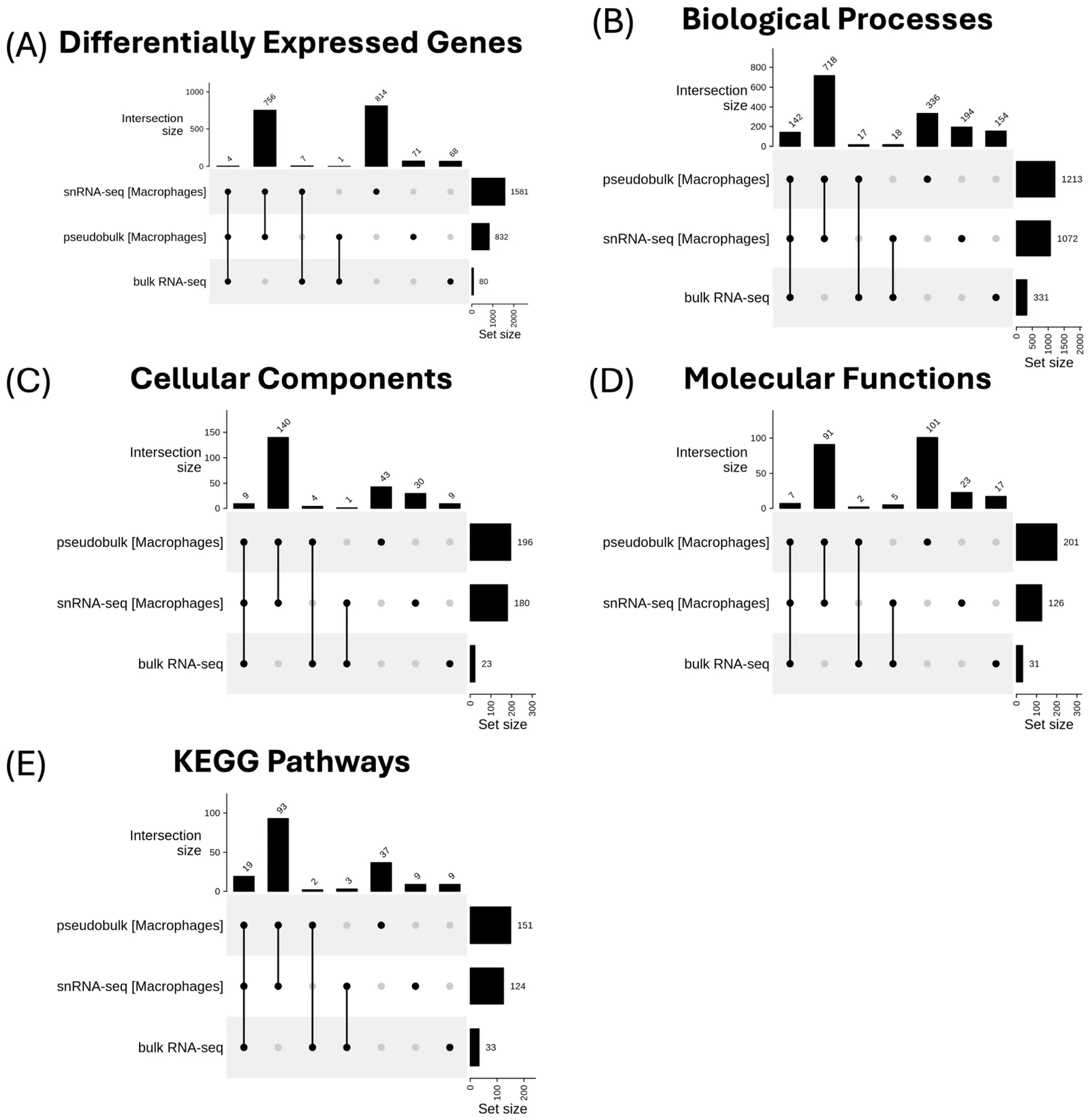
Comparison of macrophage snRNA-seq, macrophage pseudobulk, and bulk RNA-seq results across differential expression and functional enrichment analyses. UpSet plots showing the overlap between differentially expressed genes or enriched terms identified from macrophage snRNA-seq, macrophage pseudobulk, and bulk RNA-seq datasets. Vertical bars represent the number of shared or unique genes/terms, while horizontal bars indicate the total number of genes/terms in each dataset. Connected black dots indicate intersections between datasets. (**A**) Differentially expressed genes (DEGs). (**B**) Gene Ontology (GO) Biological Process enrichment analysis. (**C**) Gene Ontology (GO) Cellular Component enrichment analysis. (**D**) Gene Ontology (GO) Molecular Function enrichment analysis. (**E**) Kyoto Encyclopedia of Genes and Genomes (KEGG) pathway enrichment analysis.

To assess the consistency between macrophage snRNA-seq, macrophage pseudobulk, and bulk RNA-seq datasets, we compared differentially expressed genes (DEGs) and functional enrichment results using UpSet plots (Figure 14). A substantial overlap was observed in DEGs between macrophage snRNA-seq and macrophage pseudobulk, whereas only 12 out of 80 DEGs of bulk RNA-seq overlapped with DEGs in the other two analyses. Functional enrichment analysis demonstrated a high degree of consistency between macrophage snRNA-seq and macrophage pseudobulk across all enrichment categories. Macrophage snRNA-seq and macrophage pseudobulk shared approximately 850 biological processes (BPs), 149 cellular components (CCs), 98 molecular functions (MFs), and 112 KEGG pathways. Although bulk RNA-seq identified substantially fewer enriched terms, a large proportion of these overlapped with the macrophage-specific analyses. Specifically, 177 out of 331 enriched biological processes, 14 out of 23 cellular components, 14 out of 31 molecular functions, and 24 out of 33 KEGG pathways identified by bulk RNA-seq were also detected in either macrophage snRNA-seq or macrophage pseudobulk. These results indicate that macrophage pseudobulk closely recapitulates the functional enrichment patterns observed in macrophage snRNA-seq, while bulk RNA-seq captures fewer enriched terms but retains considerable agreement at the pathway and functional levels.

## 3. Discussion

This study demonstrates that TC1 tumors engrafted into mice with systemic MCT2 reduction exhibit increased growth compared with controls. To study tumor cell heterogeneity and seek cues to its underlying mechanisms, we performed snRNA-seq on whole tumor samples as well as bulk RNA-seq on isolated macrophages from the same tumors. These complementary approaches allowed for the exploration of differences in cellular heterogeneity between the tumors in the two experimental animal groups. Through snRNA-seq, we identified 12 distinct subpopulations of tumor cells and components of the TME, which revealed increased heterogeneity in KO tumors. We used MCT2 transgenic mice with an identical genetic background, diet, and housing environment to study the effect of the tumor, and therefore, the findings point to the far-reaching impact of MCT2 in tumor biology. Given that interactions between tumor cells and the TME can drive tumor progression and metastasis, and a scarcity of information is available on this issue, we analyzed the transcriptomes of lung tumor cells and their TME using both bulk and single-nucleus approaches [21,29]. In this study, we also leveraged the advantages conferred by comparison of snRNA-seq and bulk RNA-seq coupled with large-scale bioinformatics analyses to clarify the heterogeneity and convergence of tumors in the presence or absence of MCT2. The novelty of this study lies in the single-nucleus transcriptomic analysis of MCT2-reduction tumor microenvironment remodeling in lung cancer. While MCT2 has been studied in metabolic transport, its cell-type-specific roles within the tumor microenvironment remain poorly defined. Our snRNA-seq analysis identifies distinct immune and stromal cell populations, altered developmental trajectories, and extensive ligand–receptor communication changes associated with MCT2 reduction, indicating that MCT2 regulates tumor progression through metabolic-immune interactions within the TME.

### 3.1. MCT2

MCTs are important regulators of cellular bioenergetics and, among other roles, they primarily govern the transport of pyruvate and lactate across cellular membranes [30]. In addition, MCT2 appears to be involved in the negative regulation of cell motility and locomotion processes that help limit tumor cell invasion and metastasis in lung cancer [31,32]. Our single-nucleus RNA sequencing analysis identified 12 distinct cell populations in tumors from MCT2 CO and MCT2 KO mice following TC1 cell injection. Overall, the major cell types were preserved between groups, indicating that MCT2 deletion did not substantially alter tumor cellular composition. However, differences in top differentially expressed genes suggest changes in cellular function and activation states.

In our prior work, we showed that MCT2 knockout is associated with elevated lactate levels compared to controls, supporting a role for MCT2 in regulating lactate homeostasis [6]. Although lactate was not measured directly within tumor tissue in the present study, these findings suggest that altered lactate handling may also occur within the tumor microenvironment. Given the established role of lactate as an immunomodulatory metabolite, increased accumulation could contribute to the immunosuppressive features we observe, including M2-like macrophage polarization. Lactate is known to suppress effector immune cell function and promote alternative macrophage activation, which may in turn facilitate tumor progression. Future work using spatially resolved or tumor-specific metabolomic approaches will be important to determine how MCT2 deletion affects lactate distribution within the tumor microenvironment and to more directly link metabolic changes to immune phenotypes.

Lactate accumulation within the TME, regulated in part by monocarboxylate transporters, is increasingly recognized as a key driver of immune suppression. Elevated lactate levels inhibit effector T cell and NK cell function, while promoting M2-like macrophage polarization and suppressive cytokine programs [33]. These effects are particularly relevant to immune checkpoint blockade, which relies on functional cytotoxic lymphocytes. Furthermore, lactate influences macrophages through both receptor-mediated and intracellular pathways, mimicking hypoxia and reshaping gene expression [34]. It promotes M2-associated markers like arginase-1 and VEGF while dampening inflammation, reducing the ability of macrophages to adopt an M1 (tumor-fighting) phenotype. Given that MCT2 contributes to cellular lactate transport, its systemic deletion may affect not only tumor cell metabolism but also immune cell function and metabolic crosstalk within the TME. This raises the possibility that the observed phenotypes are linked to altered immunoregulatory pathways that could influence therapeutic response. We have incorporated recent studies to support this framework and to highlight the potential of targeting metabolic pathways to improve immunotherapy outcomes [35,36,37].

Glucose levels are significantly lower in human tumors than in normal tissues [38]. In fact, glucose metabolism is a key energy source for macrophages [39,40], fueling ATP production through glycolysis and oxidative phosphorylation (OXPHOS) [41]. As such, both glycolysis and OXPHOS are essential for ATP production during immune responses. MCT2 contributes to this process by transporting lactate into mitochondria; consequently, the reduction of MCT2 disrupts mitochondrial metabolism and suppresses colorectal cancer progression [42]. In the snRNA-seq data, a large proportion of genes tied to glycolysis, the TCA cycle, and OXPHOS were all dysregulated in the KO tumors compared to controls, forming a tightly connected module, suggesting MCT2 helps coordinate energy metabolism in the tumor microenvironment to restrict its growth.

### 3.2. Macrophages

Macrophages are central players in the tumor microenvironment by influencing inflammation, immune regulation, angiogenesis, invasion, and metastasis. They also enhance the effects of chemo-, radio-, and immunotherapy by promoting tumoricidal activity and adaptive immune responses [4]. As previously alluded, cancer cells, driven by oncogenes, rely heavily on glycolysis—even in oxygen-rich conditions—a phenomenon known as the Warburg effect [43]. This metabolic shift fuels rapid growth and survival by providing essential biosynthetic precursors [43]. Excess lactate produced by glycolysis is released into the tumor microenvironment, where it promotes immunosuppressive M2 macrophage polarization, thereby aiding tumor progression [44]. Macrophages exhibit functional plasticity along a spectrum from pro-inflammatory M1-like to anti-inflammatory M2-like states [45,46]. In most solid tumors, TAMs skew toward the M2-like phenotype, supporting tumor growth. This polarization is metabolically driven: M1 macrophages depend on glycolysis and the pentose phosphate pathway, while M2 macrophages rely on oxidative phosphorylation and fatty acid oxidation [47]. Hypoxia, lactate buildup, and nutrient scarcity further shape TAM states and functions. In lung cancer, TAMs are lactate dependent and show spatial and functional heterogeneity, with distinct metabolic and immunosuppressive traits [48,49,50,51,52,53]. These findings position TAMs as both contributors to tumor progression and promising therapeutic targets. Strategies to reprogram M2-like TAMs toward an M1-like state or disrupt their metabolic support systems could enhance anti-tumor immunity and improve lung cancer outcomes. It is important to note that larger tumors may develop hypoxic microenvironments that promote M2-like macrophage polarization. The subcutaneous flank tumor model reflects a localized tumor setting rather than a systemic cancer model. As such, the observed increase in tumor size likely arises from local microenvironmental changes, including hypoxia, which is known to drive M2-like polarization. However, because MCT2 deletion is systemic, it is difficult to determine whether the macrophage phenotype results from cell-intrinsic effects or is secondary to increased tumor burden and associated microenvironmental factors.

Recent studies have demonstrated that macrophage lipid metabolic reprogramming promotes immunosuppressive M2-like phenotypes, increases the accumulation of C1QC+ macrophages, and contributes to T-cell exhaustion within the tumor microenvironment [54,55,56,57]. Because macrophages were among the most affected immune cell populations in our snRNA-seq analysis, these findings support the possibility that host MCT2 reduction may alter macrophage metabolic programming and immune function. Future studies using macrophage-specific metabolic analyses and conditional MCT2 knockout models will be necessary to establish these mechanisms. Furthermore, lipid metabolism is extensively reprogrammed within the TME to support tumor growth and survival [58]. Alterations in lipid uptake, synthesis, transport, and catabolism reshape the TME, enabling tumor cells to adapt to changing nutrient availability and promote proliferation, invasion, and metastasis [59]. Moreover, tumor cells can induce lipid accumulation in TAMs, promoting their polarization toward an immunosuppressive M2-like phenotype [60].

### 3.3. Bulk RNA-Seq Macrophages

Bulk RNA-seq of purified tumor-associated macrophages revealed significant transcriptional and metabolic reprogramming within the tumor microenvironment. Differentially expressed genes were enriched in pathways related to glycolysis, carbon metabolism, amino acid biosynthesis, lysosomal function, and lipid metabolism, highlighting the importance of metabolic adaptation in macrophage function. Enrichment of HIF-1, MAPK, PPAR, and TGF-β signaling pathways suggests that tumor-associated macrophages respond to hypoxic and inflammatory cues that promote pro-tumorigenic activities [61,62]. Gene Ontology analysis further identified processes involved in immune regulation, leukocyte migration, lipid transport, and extracellular matrix organization, indicating roles in both immune modulation and tissue remodeling.

Together, these findings demonstrate that tumor-associated macrophages undergo coordinated metabolic and functional changes that may support tumor progression. Targeting these metabolic pathways could represent a promising strategy to modulate macrophage function and enhance anti-tumor immunity.

### 3.4. Comparisons of snRNA-Seq and Macrophage Bulk RNA-Seq

To identify conserved transcriptional programs in TAMs, we compared macrophages in snRNA-seq, pseudobulk, and bulk RNA-seq datasets. Comparison of gene set enrichment results from both datasets revealed significantly enriched KEGG pathways including MAPK and Wnt signaling pathways. These pathways are well-established regulators of inflammatory signaling, immune suppression, and cell–cell communication and have been implicated in cancer progression, invasion, metastasis, and therapeutic resistance.

We next compared all significantly enriched genes identified across all cell types in snRNA-seq clusters (clusters 0–11) with the bulk RNA-seq dataset. This analysis identified multiple shared KEGG pathways, including phagocytosis, calcium signaling, HIF-1 signaling, TGF-β signaling, glycolysis/gluconeogenesis, and carbon metabolism. These pathways are closely linked to key hallmarks of cancer, including tumor growth, adaptation to hypoxia, metabolic reprogramming, immune regulation, and metastatic progression. Notably, enrichment of glycolysis/gluconeogenesis, carbon metabolism, and HIF-1 signaling pathways suggest that TAMs undergo substantial metabolic adaptation within the hypoxic tumor microenvironment. In contrast, enrichment of phagocytosis and cell adhesion pathways highlights their involvement in tumor-immune interactions and communication with surrounding stromal and malignant cells.

Together, these findings indicate that TAMs exhibit conserved transcriptional, signaling, and metabolic programs characterized by pathways involved in inflammation, hypoxia response, metabolism, and cell adhesion. Such functional reprogramming within the lung tumor microenvironment may contribute to tumor progression, immune suppression, and therapeutic resistance, highlighting potential opportunities for macrophage-targeted interventions in lung cancer.

### 3.5. Concordance and Complementarity of snRNA-Seq and Bulk RNA-Seq

The comparisons of single-nucleus and bulk RNA-seq datasets provide several important advantages. First, it enables validation across independent transcriptomic platforms. While snRNA-seq identifies transcriptional programs at single-nucleus resolution, bulk RNA-seq provides deeper and more robust measurements of gene expression across the entire macrophage population. The overlap of enriched genes and pathways between these datasets supports the biological relevance of the identified macrophage programs and reduces the likelihood that the findings are driven by technical artifacts.

Second, comparing these datasets links cellular heterogeneity to functional biology. Single-nucleus analysis reveals distinct macrophage subpopulations and their unique transcriptional states, whereas bulk RNA-seq captures the overall functional landscape of the macrophage compartment. The concordance between the two approaches demonstrates that pathways identified within individual macrophage subsets are also reflected at the population level.

Finally, the shared genes and pathways identified across sequencing approaches represent conserved TAM signatures that likely reflect fundamental macrophage functions within the tumor microenvironment. Their reproducible enrichment across independent datasets strengthens confidence in their biological significance and highlights these conserved programs as priority candidates for future mechanistic studies and macrophage-targeted therapeutic strategies in lung cancer.

### 3.6. Methodological Differences and Limited Concordance Between Transcriptomic Approaches

The limited overlap between datasets likely reflects differences in methodology and cellular composition. The bulk RNA-seq dataset was generated from CD11b^+^-enriched tumor immune cells, whereas the snRNA-seq dataset included multiple immune and stromal cell populations. In addition, bulk RNA-seq captures averaged expression across cells, while snRNA-seq provides cell-type specific profiles with lower transcript detection per nucleus. However, our findings support the study objective of determining how MCT2 reduction affects tumor composition and signaling within the tumor microenvironment. snRNA-seq analysis revealed changes in immune and stromal populations, developmental trajectories, and intercellular communication networks. These changes are consistent with the increased tumor growth observed in MCT2-reduction mice and suggest that MCT2 helps regulate metabolic–immune interactions in lung tumors.

### 3.7. Study Strengths and Limitations

This study has several strengths, including the use of complementary in vivo, transcriptomic, and functional approaches to investigate the role of host MCT2 in lung tumor progression. However, several limitations should be acknowledged. First, our experiments were performed only in young male mice using a subcutaneous TC1 tumor model with systemic, inducible MCT2 reduction, limiting direct translation to human lung cancer and preventing identification of the specific host cell populations responsible for the observed phenotypes. Second, although our findings suggest that MCT2 influences tumor growth, invasion, and macrophage remodeling, the underlying mechanisms remain only partially defined. The definitive discrimination of implanted TC1 cells from host mesenchymal/stromal cells would require additional lineage-tracing or copy-number-based analyses. Although these experiments would provide valuable confirmation, they are beyond the scope of the present study, which was primarily designed to investigate the effects of MCT2 deficiency on the tumor microenvironment and macrophage-associated metabolic remodeling rather than to comprehensively define tumor-cell lineage. Our findings demonstrate that MCT2 reduction is associated with increased tumor growth and altered macrophage transcriptional programs; however, additional functional and cell-type-specific studies are required to establish whether these relationships are causal.

The snRNA-seq analysis identified changes in cellular composition, gene expression, and predicted cell–cell interactions, but these observations are largely descriptive and require functional validation. Macrophages emerged as one of the most affected cell populations, warranting further investigation of MCT2-reduction metabolic reprogramming, immune function, and polarization using cell-type-specific knockout models and targeted functional studies. Third, comprehensive metabolomic analyses were not performed, despite previous evidence of altered lactate metabolism in MCT2-reduction mice. Finally, the inducible knockout strategy cannot completely exclude potential effects of tamoxifen administration or Cre recombinase expression, and we did not examine whether increased MCT2 activity promotes TC1 tumor progression without tamoxifen-treated Cre-negative littermates or other treatment/genotype controls. Future studies incorporating both sexes and multiple age groups, comprehensive metabolic profiling, appropriate genetic controls, gain-of-function approaches, and cell-type-specific MCT2 models will further define the mechanisms by which host MCT2 regulates tumor progression.

### 3.8. Future Directions for Defining MCT2-Dependent Macrophage Functions

Although our study was not designed to directly assess macrophage metabolism or polarization, our transcriptomic findings suggest that host MCT2 reduction may influence macrophage function through metabolic reprogramming. Given the role of MCT2 in lactate and pyruvate transport, the reduction of MCT2 may alter metabolic signaling pathways that regulate macrophage activation and immune responses, thereby contributing to tumor progression. However, our data does not directly demonstrate changes in macrophage metabolic programs or polarization states. Future studies measuring pyruvate, extracellular pH, and metabolic flux, together with macrophage-specific metabolic profiling, functional assays, and cell-type-specific MCT2 knockout models, will be necessary to define the mechanisms by which MCT2 regulates macrophage metabolism and immune function within the tumor microenvironment. Although preliminary findings indicate potential benefit, this approach has not yet achieved adequate clinical validation in patients with lung cancer. Further large-scale, rigorously designed studies are required to establish its safety, efficacy, and clinical utility.

## 4. Materials and Methods

### 4.1. Animal

The conditional MCT2 (Slc16a7) knockout mouse model associated with C57BL/6 background (project #386-MCT2) was produced at Ingenious Targeting Laboratory (Ronkonkoma, NY, USA) by intercrossing MCT2loxP mice with mCre-Tg mice (The Jackson Laboratory stock #008463, Bar Harbor, ME, USA) to create MCT2loxP/loxP; Cre+ mice (MCT2 KO) [6]. At 8 weeks of age, the MCT2 KO mice were injected with tamoxifen (0.18 mg/g body weight) once daily for five consecutive days, followed by subcutaneous injection of TC1 cells (100,000 cells/mouse) two days later. Genotyping was performed using MCT2loxP mouse genotyping primers (MCT2-R: CTA TCA CGC TGT TGC TGT AAG A; MCT2-F: GAC TCC CTT CTC CCA TCT CAG), yielding 319 bp (wildtype) and 380 bp (Flox+/+) fragments for MCT2. Likewise, further genotyping work was carried out with Cre+ genotyping primers (8463-1: AAA GTC GCT CTG AGT TGT TAT (mCre wildtype forward); 8463-2: GGA GCG GGA GAA ATG GAT ATG (mCre wildtype reverse) yielding 650 bp (wildtype) and 825 bp (Cre+/+) fragments for Cre recombinase. At 2 months of age, MCT2 positive males and females (2 months old) were bred. In this study, tamoxifen-treated mice are referred to as MCT2 knockout (KO), whereas vehicle-treated mice are designated as control (CO). All procedures were approved by the Institutional Animal Care and Use Committee (IACUC #9586 and #9601, 10 November 2020) at the University of Missouri.

### 4.2. Tumor Cell Lines

Mouse epithelial lung tumor cells, which are also known as TC1 cells (ATCC #, CRL-2785), were procured from American Type Culture Collection (Manassas, VA, USA) and cultured in a 37 °C humidified incubator containing 5% CO_2_ and 95% air [63,64]. The TC1 cells were cultured in RPMI medium provided with 2 mM L-glutamine, 10 mM HEPES buffer, 1 mM sodium pyruvate, 0.1 mM non-essential amino acids, 100 U/mL penicillin, 100 µg/mL streptomycin, 10% fetal bovine serum (FBS), and 0.4 mg/mL geneticin (Life Technologies, Grand Island, NY, USA).

### 4.3. Tamoxifen and Subcutaneous Lung Tumor Model

Tamoxifen dissolved in sunflower oil (20 mg/mL) was administered and injected into MCT2 transgenic mice at a dosage of 180 µg/g body weight once daily for five consecutive days. This dosing regimen was selected based on prior optimization studies indicating its effectiveness in reducing MCT2 expression [6]. Mice treated with tamoxifen, denoted as KO (*n* = 10), and those receiving vehicle without tamoxifen as controls, CO (*n* = 10), were engrafted subcutaneously with TC1 cells (100,000 cells in 200 µL PBS) by subcutaneous injection into the lower right flank. Tumor volumes were observed and measured for length and width three times a week using an electronic caliper. Twenty-four days after TC1 cell injections, mice were sacrificed, and both blood and tumors were collected [63,64].

### 4.4. Tumor Growth

Mice were monitored daily and assessed using the body condition scoring (BCS) system. Tumor growth was examined three times per week using digital calipers. Animals in the solid tumor model were euthanized if tumors ulcerated, reached 15 mm (1.5 cm) in diameter, or if the animals exhibited signs of discomfort or distress, including impaired eating, drinking, grooming, or excessive weight loss. All remaining animals were euthanized on day 24, after which tumors were excised for downstream analyses, including blood collection and tissue harvesting. All mice were euthanized using 3% carbon dioxide (CO_2_) as a primary method and followed by cervical dislocation as a secondary method to guarantee death. Animals were monitored daily and euthanized immediately if any humane endpoint criteria, including the maximum tumor diameter, ulceration, impaired mobility, or signs of distress, were reached. Tumor volumes were measured longitudinally during tumor growth, whereas all other tumor measurements were obtained only at the time of animal sacrifice. Therefore, tumor volume is the only outcome that requires repeated-measures analysis. Tumor growth was monitored by 10 mice per group to ensure adequate statistical power for longitudinal analysis. Tumor volume was calculated using the formula Volume (mm^3^) = (length × width^2^)/2, where length represents the longest diameter and width is the perpendicular diameter.

### 4.5. Macrophage Isolation and Flow Cytometry

Subcutaneous tumors from KO and CO mice were excised immediately, finely minced, and enzymatically digested using 2 mg/mL collagenase 4 in RPMI medium supplemented with 0.1% BSA (1 g tumor tissue/10 mL medium), then gently agitated for 45 min in a 37 °C incubative environment. Following the digestion step, the tissues were strained through a nylon mesh (70 μm) and rinsed with buffer containing high salt concentration and 1% bovine serum albumin (BSA).

Macrophages (CD11b^+^) cells were purified from the cell suspension by following the EasySep Mouse CD11b Positive Selection Kit II (STEMCELL, #18970) protocol provided by the manufacturer (Vancouver, BC, Canada). Next, both M1 marker CD86 (#105028, San Diego, CA, USA) and an M2 marker CD206 (#141708, San Diego, CA, USA) were targeted in TAM flow cytometric analysis. In preparation, Before staining, all dyes and antibodies were protected from light and maintained at the appropriate temperature. When ready, a Canto II instrument (BD Biosciences, San Jose, CA, USA) was utilized to perform flow cytometry analyses, and the output data was analyzed utilizing the Flowjo software v11.0 (Treestar Inc, Ashland, OR, USA). The results were presented as either percentages of the immediate parent population or fold changes relative to the corresponding control. Flow cytometric analysis was performed on 7–8 tumors per group because not all harvested tumors yielded sufficient numbers of viable cells following tissue dissociation and processing. Samples with inadequate cell recovery or viability were excluded according to predefined technical quality criteria, independent of the experimental outcomes.

### 4.6. Single-Nucleus RNA-Sequencing (snRNA-Seq)

The protocol utilized for isolation of nuclei from frozen mouse tumor tissues for 10x snRNA-seq differed slightly compared to the one previously described [20]. Tumor samples (200 mg of tissue/sample) were homogenized using a Dounce homogenizer (VWAR, Batavia, IL, USA) in RNase-free lysis buffer (10mM Tris-HCl, 10mM NaCl, 3mM MgCl2, 0.1% NP40) on ice, followed by lysis at 4 °C for 5 min. Lysates were then filtered through a 40 μm nylon filter (CellTreat, Ayer, MA, USA) and collected into a 50 mL conical tube (Corning, NY, USA). The filter was subsequently rinsed with 3 mL of wash buffer (146 mM NaCl, 1 mM CaCl_2_, 21 mM MgCl_2_; 10 mM Tris-HCl pH 7.5) with or without 0.2 U/μL RNase Inhibitor (ThermoFisher Scientific, Cincinnati, OH, USA), and the rinse was combined with the initial filtrate. The resulting flow-through was centrifuged at 500× *g* for 5 min at 4 °C. Following centrifugation, supernatants were discarded, and the nuclear pellets were resuspended in 50–200 μL PBS (pH 7.4) (ThermoFisher Scientific, Cincinnati, OH, USA) containing 0.02% BSA, with or without RNase inhibitor (0.2 U/µl RNase inhibitor). To minimize contamination from ambient RNA, selected nuclear pellets of samples underwent 1–3 additional washes in 5 mL PBS supplemented with 0.02% BSA prior to final resuspension. Nuclei were quantified using an automated cell counter, Countess II Automated Cell Counter (Life Technologies, Carlsbad, CA, USA), to determine the appropriate input volume for downstream single-nucleus library preparation as guided in the 10x Chromium Single Cell Library protocol. Single-nucleus libraries were generated using the Chromium Next GEM Single Cell 3′ Kit v3.1 with Chip G (10x Genomics, Pleasanton, CA, USA). Approximately 4000 nuclei per sample were loaded into the Chromium Controller to produce gel bead-in-emulsions (GEMs). cDNA synthesis and library construction were carried out according to the manufacturer’s protocol. Final libraries were sequenced on an Illumina NextSeq platform with a target of around 25,000 reads per cell.

#### 4.6.1. Single-Nucleus RNA-Seq Integrative Analysis

The snRNA-seq Bioinformatics analysis was performed on six datasets corresponding to three KO and three CO conditions. Raw sequencing read quality of all datasets was assessed using the FastQC tool v0.12.0 and the MultiQC tool v1.28 [65] to ensure high-quality input for downstream processing. Reads were then aligned to the mouse reference genome (GRCm38) and quantified using Cell Ranger v3.0.2. Upon completion, SoupX v1.6.2 [66] was utilized to remove ambient RNA contamination and generate six matrices suitable for analysis with the Seurat v5.2.1 package [67] in the R programming language v4.3.1. The Read10x function in the Seurat package was employed to import the matrices, followed by the Create Seurat Object function to generate individual Seurat objects for each sample. During the quality control step, nuclei were only retained if they expressed between 100 and 7500 genes and had less than 20% of reads mapped to mitochondrial genes to minimize the inclusion of low-quality or dying cells. Subsequently, scDblFinder v1.24.0 [68] was utilized to detect and remove doublets. The metrics showing before and after the quality control step are also included in Table S21. The filtered datasets were then normalized and variance-stabilized using the SCTransform function [69,70] and subsequently integrated across all samples using the Harmony algorithm [71] to correct for batch effects and inter-sample variability. Following integration, a shared nearest neighbor (SNN) graph was constructed using the FindNeighbors function, and clusters were identified with the FindClusters function. Dimensionality reduction was performed using both the RunTSNE and RunUMAP functions to enable visualization of the cellular heterogeneity and clustering in two-dimensional space. The snRNA-seq datasets have been submitted to the Expression Omnibus database (GEO) (snRNA-seq Data GEO ID: GSE338088).

#### 4.6.2. Single-Nucleus RNA-Seq Differential Expression Analyses

Differential expression analyses were performed using the Seurat FindAllMarkers and FindMarkers functions provided by the Seurat package. The Seurat FindAllMarkers function was utilized to compare cells in each cell type to the cell types of the rest of the cell types combined to identify significant differentially expressed genes (DEGs) in each cell type with filtering thresholds of an absolute log_2_ fold change ≥ 0.58 and adjusted *p*-value ≤ 0.05. On the other hand, the FindMarkers function was utilized to compare all the cells between the KO over CO conditions and the cells in each cell type between the KO over CO conditions. The global level approach can export significant DEGs overall for a mix of cell types, and the cell type specific approach can roll out significant DEGs specific to cell types (filtered by an absolute log_2_ fold change ≥ 0.58 and adjusted *p*-value ≤ 0.05). Differential expression analysis of snRNA-seq data was performed using the Wilcoxon rank-sum test implemented in Seurat. *p*-values were adjusted for multiple testing using the Benjamini–Hochberg method. The unit of analysis for this test was individual nuclei. To address the concern regarding sample-to-sample heterogeneity, we performed a pseudobulk differential expression analysis as well.

#### 4.6.3. Single-Nucleus RNA-Seq Cell Type Annotation

Significant differentially expressed genes (DEGs) identified from the Seurat FindAllMarkers function were used as inputs for downstream cell type annotation. The cell type annotation was performed using the Single Cell Signature Annotator (SCSA) tool (https://github.com/bioinfo-ibms-pumc/SCSA (accessed on 1st September 2025)) [72] which leverages a curated set of reference markers from the Cell Marker database v2 [73]. To ensure robust and biologically meaningful annotations, filtering thresholds including a *p*-value cutoff of 0.05 and an absolute minimum fold change of 1.5 were applied. These criteria helped prioritize high-confidence marker genes for accurate assignment of cell identities across clusters.

Our group also extracted the top 15 differentially expressed marker genes from Seurat FindAllMarkers results and put them into Table S22 to assist in cell type annotations using PanglaoDB [74]. On PanglaoDB, the top 15 marker genes of each cell type were input to search for cell types related to the lung separately.

Moreover, a selected set of marker genes related to each cell type was also prepared (Table S23). These marker genes were plotted in dot plots to visualize sample-combined and sample-wise expression patterns to support cell type annotations. The sample combined dot plots are in Figures S6–S8, while the sample-wise dot plots are in Figures S9–S20.

#### 4.6.4. Single-Nucleus RNA-Seq Gene Set Enrichment Analyses

The gene set enrichment analyses for the single-nucleus RNA-seq (snRNA-seq) were performed using clusterProfiler v4.16.0 [75]. The input significant differentially expressed gene lists to clusterProfiler were from the global level snRNA-seq differential expression analysis and the cell type level snRNA-seq differential expression analysis. The enrichment outputs contained biological processes (BPs), cellular components (CCs), molecular functions (MFs), KEGG pathways, and Reactome pathways. To obtain significant enrichment results, a filtering threshold of q-value ≤ 0.05 was used in all the enrichment analyses (Tables S24 and S25).

#### 4.6.5. Single-Nucleus RNA-Seq Cell Trajectory and Lineage Analyses

Cell trajectory and lineage analyses were performed independently for the MCT2 knockout (KO) and MCT2 control (CO) datasets using Slingshot v2.16.0 [76]. For each condition, the Seurat object was converted to a SingleCellExperiment object for compatibility with Slingshot. Lineage inference and pseudotime estimation were performed using the slingshot function with the Uniform Manifold Approximation and Projection (UMAP) embedding and annotated cluster identities as input. Slingshot inferred putative lineage relationships among cell populations and assigned pseudotime values to individual nuclei to enable reconstruction of lineage trajectories and cell-state progression within each condition. This analysis was used to compare the inferred lineage architecture and pseudotime organization between MCT2 KO and CO samples.

#### 4.6.6. Single-Nucleus RNA-Seq Cell–Cell Interaction Analyses

The cell–cell communication analysis was conducted using CellChat v2.1 [77] with its built-in mouse database. The expression data, metadata, and cell type annotations in the Seurat object were extracted and treated as inputs to the createCellChat function to create a CellChat object for each condition separately. The intermediate steps in the analysis process include identifying over expressed genes and interactions using the identifyOverExpressedGenes and identifyOverExpressedInteractions functions, computing for communication probability and strength between cell types using the computeCommunProb function, estimating communication probability on signaling pathway level for all ligands and receptors with the computeCommunProbPathway function, aggregating and summarizing the numbers of communications and probabilities using the aggregateNet function, as well as computing for the network centrality scores for all inferred communication networks using the netAnalysis_computeCentrality function. For visualization, the netVisual_circle function was mainly utilized to plot the interaction networks between different cell type clusters. The netVisual_heatmap function was used to plot the heatmaps for incoming and outgoing signaling of different signaling groups in different cell types. Moreover, the netVisual_chord_gene function was utilized to plot the ligand and receptor interaction networks for incoming and outgoing signaling in different cell types. The cell–cell interaction analyses were performed for KO and CO comparison as well as comparisons of 3 KO samples and 3 CO samples.

#### 4.6.7. Pseudobulk Analysis Using Single-Nucleus RNA-Seq Data

Single-nucleus RNA-seq data were aggregated into pseudobulk expression profiles using Seurat AggregateExpression function to generate pseudobulk data for each cell type with three knockout (KO) samples and three control (CO) samples. Principal component analysis (PCA) was performed on the pseudobulk data, which revealed a clear separation pattern between the three KO samples and 3 control samples (Figure S21A). Differential expression analysis was then performed independently for each cell type using DESeq2 v1.46.0. As a sensitivity analysis, pseudobulk differential expression analysis was performed using biological replicates as the unit of inference. Applying thresholds of absolute log2 fold change ≥ 0.58 (corresponding to a 1.5 fold change) and adjusted *p*-value ≤ 0.05, significantly differentially expressed genes (DEGs) were identified in multiple cell types (Table S26).

#### 4.6.8. Pseudobulk Gene Set Enrichment Analysis

For each cell type, genes from pseudobulk analysis were ranked according to the DESeq2 Wald statistic, and gene set enrichment analysis was performed using the clusterProfiler v4.16.0 [75]. This approach evaluates whether sets of functionally related genes exhibit consistent expression changes, even when individual genes do not reach statistical significance. Using a significance threshold of q-value ≤ 0.05, multiple KEGG pathways were identified as significantly enriched in several cell types, which indicate that the knockout is associated with coordinated perturbations of biological processes despite the absence of statistically significant differentially expressed genes at the individual gene level (Table S27).

### 4.7. Bulk RNA-Sequencing (RNA-Seq) of Tumor Macrophages

The RNeasy Lipid Tissue Mini Kit with on-column DNase digestion (Qiagen, Valencia, CA, USA) was utilized to isolate the total RNA from TAMs following the manufacturer’s instructions [78]. Next, the Eukaryote Total RNA Nano 6000 LabChip assay (Agilent Technologies, Santa Clara, CA, USA) on an Agilent 2100 Bioanalyzer (Thermo Fisher Scientific, Waltham, MA, USA) was utilized to evaluate the RNA integrity and quality. The NanoDrop ND-1000 UV−vis spectrophotometer (NanoDrop Technologies, Wilmington, DE, USA) was also used to measure absorbance at 260 nm for RNA concentration. Upon completion, Illumina’s TruSeq Stranded mRNA LT library preparation protocol (Illumina Inc., San Diego, CA, USA) was followed to prepare Poly-A enriched mRNA-Seq libraries using 0.5 µg of total RNA. Briefly, poly-A RNA was isolated and converted into cDNA libraries suitable for sequencing (Illumina, Inc., San Diego, CA, USA), and the sequencing activity was carried out on an Illumina HiSeq 2500 platform (Illumina, Inc., San Diego, CA, USA). The bulk RNA-seq datasets have been submitted to the Expression Omnibus database (GEO) (Bulk RNA-seq Data GEO ID: GSE338086).

#### 4.7.1. Bulk RNA-Seq Analysis

A total of six raw RNA-seq datasets consisting of the KO and CO conditions with three replicates each were analyzed. All RNA-seq datasets were screened for quality control using the FastQC tool v0.12.0 and the MultiQC tool v1.28 [65]. For alignment, the raw reads were aligned to the mouse reference genome (GRCm38) using the STAR tool v2.7.11b [79,80]. Next, the count matrix was generated using Subread featureCounts v2.1.1 [81,82], and the differential expression analysis was completed using the DESeq2 tool v1.18.1 [82,83] to output the differential expression gene list. The genes in the differential expression gene list were filtered using q-value ≤ 0.05 and absolute log_2_ fold change ≥ 0.58 to obtain significant differentially expressed genes (DEGs). Apart from that, principal component analysis (PCA) was performed on the bulk RNA-seq data as well and revealed a clear separation pattern between three KO samples and three control samples (Figure S21B).

#### 4.7.2. Bulk RNA-Seq Gene Set Enrichment Analysis

The gene enrichment analysis was performed using clusterProfiler v4.16.0 [75] with the significant differentially expressed genes (DEGs) from the bulk RNA-seq analysis. The outcomes of the enrichment analysis consist of significant biological processes (BPs), cellular components (CCs), molecular functions (MFs), KEGG pathways, and Reactome pathways filtered by q-value ≤ 0.05.

## 5. Conclusions

Our study suggests that MCT2 contributes to shaping the tumor immune microenvironment and constraining the progression of TC1 lung carcinoma in a murine model. Reduced MCT2 expression was associated with significant changes in cellular composition, gene expression patterns, and signaling pathways. snRNA-seq analysis further indicated that MCT2 reduction alters immune cell behavior, as well as metabolic and immune regulatory processes. In addition, distinct ligand–receptor interaction patterns observed in MCT2-reduction tumors suggest that MCT2 influences intercellular communication within the tumor microenvironment. Overall, these findings support a role for MCT2 in regulating metabolic–immune crosstalk during lung tumor progression. However, further studies, particularly those involving clinical validation, are needed to clarify the underlying mechanisms and assess the therapeutic potential of targeting MCT2.

## Figures and Tables

**Table 1:** Cell types and their top five marker genes in MCT2 knockout (MCT2 KO) and MCT2 control (MCT2 CO) mice following flank injection of TC1 cells (Figure 2C,D).

Cluster	MCT2 CO Marker Genes	MCT2 KO Marker Genes
0 [Macrophages]	Gm5150, Abcg3, Cadm1, Mrc1, Cd300lf	Rnf180, Adap2, Blnk, Mrc1, Gm5150
1 [Mesenchymal Stromal Cells]	Dock3, Adamtsl5, Dkk2, Cpeb1, Ppp1r9a	Ank3, Adcy8, Btbd11, Chn1, Dkk2
2 [Cycling Mesenchymal Stromal Cells]	Ankle1, Cep55, Depdc1a, Kif18a, Pif1	Neil3, Knl1, Kif4, Nek3, Kif18a
3 [Fibroblasts]	Pde10a, Frmd5, Gpm6b, Col12a1, Kcnk10	Frmd5, Pde10a, Sned1, Gpm6b, Gm2115
4 [Mesenchymal Cells]	Anxa5, Pgam1, Pgk1, Crabp1, Alyref	Eef1a1, Actb, Actg1, Ppia, Rps16
5 [T/NK Cells]	Ikzf3, Itk, Tox, Themis, Prkcq	Themis, Tox, Skap1, Tmem163, Bcl11b
6 [Interferon-responsive Mesenchymal Stromal Cells]	Ifit3b, Rsad2, Ifit1, Ifit3, Oasl1	Rsad2, Ifit1, Oasl1, Ifit3b, Ifit3
7 [Endothelial Cells]	Ldb2, Cdh13, Ptprb, Adgrf5, Fbxl7	Ldb2, Prex2, Emcn, Adgrl4, Ptprb
8 [Activated/Cycling Cells]	Hist1h2ac, Atf3, Egr4, Ppp1r15a, Hist1h2bc	Ier2, Dnajb1, Il31ra, Jun, Egr1
9 [ECM-remodeling Fibroblasts]	Tshz3, Brinp3, Tshz2, Robo1, Kcnma1	Chsy3, Kcnma1, Robo1, Epha3, Col11a1
10 [Adipocytes]	Zbtb16, Cd36, Mgll, Sntb1, Tshr	Tshr, Cd36, Zbtb16, Mgll, Sorbs1
11 [Skeletal Muscle Cells]	Ttn, Atp2a1, Kcnma1, Rbm20, Trdn	Prr33, Rbm20, Trdn, Kbtbd12, Phkg1

**Table 2:** Cell types with their top 5 marker genes integrated MCT2 knockout (MCT2 KO) and MCT2 control (MCT2 CO) mice injected with TC1 cells in the flank (Figure 3A).

Cluster	MCT2 Marker Genes
0 [Macrophages]	Gm5150, Mrc1, Abcg3, P2ry6, Fgd2
1 [Mesenchymal Stromal Cells]	Dock3, Chn1, Dkk2, Adamtsl5, Ranbp17
2 [Cycling Mesenchymal Stromal Cells]	Cep55, Depdc1a, Nek3, Kif18a, Neil3
3 [Fibroblasts]	Pde10a, Frmd5, Gpm6b, Medag, Col12a1
4 [Mesenchymal Cells]	Actb, Hspa8, Anxa5, Eef2, Pgk1
5 [T/NK Cells]	Itk, Ikzf3, Tox, Tmem163, Themis
6 [Interferon-responsive Mesenchymal Stromal Cells]	Ifit3b, Rsad2, Ifit1, Oasl1, Ifit3
7 [Endothelial Cells]	Ldb2, Prex2, Ptprb, Cdh13, Adgrl4
8 [Activated/Cycling Cells]	Hist1h2ac, Atf3, Hist2h3c1, Ppp1r15a, Hist1h2bc
9 [ECM-remodeling Fibroblasts]	Kcnma1, Epha3, Chsy3, Brinp3, Robo1
10 [Adipocytes]	Tshr, Zbtb16, Cd36, Sorbs1, Mgll
11 [Skeletal Muscle Cells]	Ttn, Trdn, Rbm20, Prr33, Atp2a1

**Table 3:** Cell types with their top 10 marker genes in MCT2 knockout (MCT2 KO) compared with MCT2 control (MCT2 CO) mice injected with TC1 cells in the flank (Figure 3B).

Cluster	Differentially Expressed Marker Genes in KO vs. CO
0 [Macrophages]	Bnc2, Ckm, Ghr, Gulp1, Itgb8, Nox4, Pak3, Setbp1, Ttn, Zfpm2
1 [Mesenchymal Stromal Cells]	Arhgap15, Atp8b4, Dock2, Elmo1, Frmd4b, Lhfpl3, Nxph1, Plxna4, Sorcs1, Unc5c
2 [Cycling Mesenchymal Stromal Cells]	Abca1, Kif26b, Lhfpl3, Nek10, Nxph1, Pde7b, Rnf150, Slco5a1, Taco1, Tmem200a
3 [Fibroblasts]	Adam12, Cacnb4, Dkk2, Ebf2, Elmo1, Lhfpl3, Prkg1, Pstpip2, Rnf150, Unc5c
4 [Mesenchymal Cells]	Arhgap15, Cadm1, Elmo1, Lrmda, Lyn, March1, Pik3ap1, Pparg, Slc8a1, Vav1
5 [T/NK Cells]	Adgrl3, Bnc2, Ghr, Hmga2, Mecom, Nfib, Pbx1, Rbms3, Sema7a, Zfpm2
6 [Interferon-responsive Mesenchymal Stromal Cells]	Adcy8, Babam2, Col12a1, Foxo3, Itgb8, Klf12, Rbpj, Tbl1x, Vps13b, Zc3h12b
7 [Endothelial Cells]	Rpl19
8 [Activated/Cycling Cells]	Gm2000, H2-D1, Ubb
10 [Adipocytes]	Sox6
11 [Skeletal Muscle Cells]	Sox6

**Table 4:** List of KEGG pathways identified in DEGs in tumor macrophages using snRNA-seq.

KEGG Pathway	DEGs in Macrophages
Chemokine signaling pathway	Akt3, Ccl2, Ccl5, Dock2, Elmo1, Gnaq, Prkcb
PI3K-Akt signaling pathway	Akt3, Col4a1, Ghr, Itga6, Phlpp1, Tnc
IgSF CAM signaling	Akt3, Cadm1, Dynll1, Prkcb, Slit2, Tubb4b
Antigen processing and presentation	Cd74, Ctsl, H2-Ab1, H2-D1, H2-Eb1, Hspa8
MAPK signaling pathway	Akt3, Hspa8, Mecom, Ppp3ca, Prkcb
Focal adhesion	Akt3, Col4a1, Itga6, Prkcb, Tnc
Cell adhesion molecule (CAM) interaction	Cadm1, H2-Ab1, H2-D1, H2-Eb1, Itga6
Rap1 signaling pathway	Akt3, Gnaq, Prkcb
ECM-receptor interaction	Col4a1, Itga6, Tnc
Hematopoietic cell lineage	H2-Ab1, H2-Eb1, Itga6
Th1 and Th2 cell differentiation	H2-Ab1, H2-Eb1, Ppp3ca
Th17 cell differentiation	H2-Ab1, H2-Eb1, Ppp3ca
TNF signaling pathway	Akt3, Ccl2, Ccl5
Ras signaling pathway	Akt3, Prkcb
Cytokine–cytokine receptor interaction	Ccl2, Ccl5
HIF-1 signaling pathway	Akt3, Prkcb
Wnt signaling pathway	Ppp3ca, Prkcb
NOD-like receptor signaling pathway	Ccl2, Ccl5

## Data Availability

The original contributions presented in this study are included in the article. Further inquiries can be directed to the corresponding author.

## Supplementary Materials

The following supporting information can be downloaded at: https://www.mdpi.com/article/10.3390/ijms27177819/s1.
