# Supplementary material for: Monocarboxylate Transporter 2 (MCT2) Reduction Is Associated with Increased Lung Tumor Growth and Alterations in the Immune Microenvironment in a Subcutaneous Tumor Model
Source: Int J Mol Sci. 2026 Aug 31;27(17):7819. doi: 10.3390/ijms27177819 (PMC13566037; doi:10.3390/ijms27177819)
Supplement: Supplementary file 1 [file ijms-27-07819-s001.zip › Supplementary Figure and Table Captions.pdf]

**Supplementary Figures:**

**Figure S1.** Identification of cluster-specific marker genes in the individual MCT2 CO and MCT2 KO single-nucleus RNA sequencing datasets. (A) Dot plot showing the top five differentially expressed marker genes for each cluster identified in the MCT2 CO dataset using the Seurat FindAllMarkers function. (B) Dot plot showing the top five differentially expressed marker genes for each cluster identified in the MCT2 KO dataset using the same approach. Dot size represents the percentage of nuclei expressing each gene, while color intensity indicates the average normalized expression level.

**Figure S2.** Cluster-specific marker gene expression in the integrated MCT2 KO and MCT2 CO single-nucleus RNA sequencing dataset. Dot plot showing the top five differentially expressed marker genes for each cluster identified using the Seurat FindAllMarkers function following integration of the MCT2 KO and MCT2 CO datasets. Dot size represents the percentage of nuclei expressing each gene, while color intensity indicates the average normalized expression level.

**Figure S3.** Slingshot pseudotime analysis of MCT2 control (CO) and MCT2 knockout (KO) snRNA-seq datasets. Four putative lineages were inferred in each condition. The inferred trajectory origins and early pseudotime organization differed between MCT2 CO and KO, indicating that MCT2 deficiency alters the initial trajectory architecture of populations.

**Figure S4.** Cell-cell communication networks based on the number of inferred interactions across individual samples. CellChat circle plots showing the inferred intercellular communication networks for each sample (Control_T1_NG, Control_T2_NG, Control_T3_NG, Knockout_T1_POS, Knockout_T2_POS, and Knockout_T3_POS). Each node represents a cell population, with node size proportional to the total number of interactions (incoming and outgoing) involving that cell type. Edges indicate predicted ligand-receptor-mediated communication between cell populations, and edge thickness is proportional to the number of inferred interactions. Edge colors correspond to the signaling source (sender) cell population. Self-loops represent autocrine signaling within the same cell population. These network diagrams provide an overview of the complexity and distribution of cell-cell communication across individual samples.

**Figure S5.** Cell-cell communication networks based on interaction strength across individual samples. CellChat circle plots illustrating the inferred cell-cell communication networks for each sample (Control_T1_NG, Control_T2_NG, Control_T3_NG, Knockout_T1_POS, Knockout_T2_POS, and Knockout_T3_POS). Each node represents a cell population, with node size proportional to the total communication strength (sum of incoming and outgoing signaling) associated with that cell type. Directed edges represent predicted ligand-receptor-mediated communication between sender and receiver cell populations, with edge thickness proportional to the overall interaction strength (communication probability). Edge colors indicate the sender cell population, and self-loops represent autocrine signaling. These networks provide a global overview of the relative intensity and distribution of intercellular communication within each sample, enabling comparison of signaling activity between control and knockout groups across time points.

**Figure S6.** Expression of canonical marker genes used for cell type annotation in the integrated single-nucleus RNA sequencing dataset. Dot plots show the expression of established marker genes for (**A**) macrophages, (B) mesenchymal stromal cells, (C) cycling mesenchymal stromal cells, and (D) fibroblasts across the identified cell clusters. Dot size represents the percentage of nuclei expressing each gene, while color intensity indicates the average normalized expression level.

**Figure S7.** Expression of canonical marker genes used for cell type annotation in the integrated single-nucleus RNA sequencing dataset. Dot plots show the expression of established marker genes for (A) mesenchymal cells, (B) T/NK cells, (C) interferon-responsive mesenchymal stromal cells, and (D) endothelial cells across the identified cell clusters. Dot size represents the percentage of nuclei expressing each gene, whereas color intensity indicates the average normalized expression level.

**Figure S8.** Expression of canonical marker genes used for cell type annotation in the integrated single-nucleus RNA sequencing dataset. Dot plots show the expression of established marker genes for (A) activated/cycling cells, (B) ECM-remodeling fibroblasts, (C) adipocytes, and (D) skeletal muscle cells across the identified cell clusters. Dot size represents the percentage of nuclei expressing each gene, whereas color intensity indicates the average normalized expression level.

**Figure S9.** Sample-wise expression of canonical macrophage marker genes. Dot plots show the expression of canonical macrophage marker genes across individual MCT2 control (CO) and MCT2 knockout (KO) samples. Dot size represents the percentage of nuclei expressing each gene, whereas color intensity indicates the average normalized expression level.

**Figure S10.** Sample-wise expression of canonical mesenchymal stromal cell marker genes. Dot plots show the expression of canonical mesenchymal stromal cell marker genes across individual MCT2 control (CO) and MCT2 knockout (KO) samples. Dot size represents the percentage of nuclei expressing each gene, whereas color intensity indicates the average normalized expression level.

**Figure S11.** Sample-wise expression of canonical cycling mesenchymal stromal cell marker genes. Dot plots show the expression of canonical cycling mesenchymal stromal cell marker genes across individual MCT2 control (CO) and MCT2 knockout (KO) samples. Dot size represents the percentage of nuclei expressing each gene, whereas color intensity indicates the average normalized expression level.

**Figure S12.** Sample-wise expression of canonical fibroblast marker genes. Dot plots show the expression of canonical fibroblast marker genes across individual MCT2 control (CO) and MCT2 knockout (KO) samples. Dot size represents the percentage of nuclei expressing each gene, whereas color intensity indicates the average normalized expression level.

**Figure S13.** Sample-wise expression of canonical mesenchymal cell marker genes. Dot plots show the expression of canonical mesenchymal cell marker genes across individual MCT2 control (CO) and MCT2 knockout (KO) samples. Dot size represents the percentage of nuclei expressing each gene, whereas color intensity indicates the average normalized expression level.

**Figure S14.** Sample-wise expression of canonical T/NK cell marker genes. Dot plots show the expression of canonical T/NK cell marker genes across individual MCT2 control (CO) and MCT2 knockout (KO) samples. Dot size represents the percentage of nuclei expressing each gene, whereas color intensity indicates the average normalized expression level.

**Figure S15.** Sample-wise expression of canonical interferon-responsive mesenchymal stromal cell marker genes. Dot plots show the expression of canonical interferon-responsive mesenchymal stromal cell marker genes across individual MCT2 control (CO) and MCT2 knockout (KO) samples. Dot size represents the percentage of nuclei expressing each gene, whereas color intensity indicates the average normalized expression level.

**Figure S16.** Sample-wise expression of canonical endothelial cell marker genes. Dot plots show the expression of canonical endothelial cell marker genes across individual MCT2 control (CO) and MCT2 knockout (KO) samples. Dot size represents the percentage of nuclei expressing each gene, whereas color intensity indicates the average normalized expression level.

**Figure S17.** Sample-wise expression of canonical activated/cycling cell marker genes. Dot plots show the expression of canonically activated/cycling cell marker genes across individual MCT2 control (CO) and MCT2 knockout (KO) samples. Dot size represents the percentage of nuclei expressing each gene, whereas color intensity indicates the average normalized expression level.

**Figure S18.** Sample-wise expression of canonical ECM-remodeling fibroblast marker genes. Dot plots show the expression of canonical ECM-remodeling fibroblast marker genes across individual MCT2 control (CO) and MCT2 knockout (KO) samples. Dot size represents the percentage of nuclei expressing each gene, whereas color intensity indicates the average normalized expression level.

**Figure S19.** Sample-wise expression of canonical adipocyte marker genes. Dot plots show the expression of canonical adipocyte marker genes across individual MCT2 control (CO) and MCT2 knockout (KO) samples. Dot size represents the percentage of nuclei expressing each gene, whereas color intensity indicates the average normalized expression level.

**Figure S20.** Sample-wise expression of canonical skeletal muscle cell marker genes. Dot plots show the expression of canonical skeletal muscle cell marker genes across individual MCT2 control (CO) and MCT2 knockout (KO) samples. Dot size represents the percentage of nuclei expressing each gene, whereas color intensity indicates the average normalized expression level.

**Figure S21.** Principal component analysis (PCA) of (A) pseudobulk snRNA-seq and (B) bulk RNA-seq samples. Each point represents one biological replicate, colored by experimental group and shaped by sample identity. PC1 and PC2 explain the indicated percentages of total variance.

**Supplementary Tables:**

**Table S1.** Differentially expressed genes (DEGs) identified in each cell cluster of the MCT2 control (CO) dataset. Cluster-specific marker genes were identified independently for each of the 12 clusters using the Seurat FindAllMarkers function by comparing each cluster with all other clusters within the MCT2 CO dataset. The table lists the significantly enriched marker genes for each cluster that were used for downstream analyses.

**Table S2.** Differentially expressed genes (DEGs) identified in each cell cluster of the MCT2 knockout (KO) dataset. Cluster-specific marker genes were identified independently for each of the 12 clusters using the Seurat FindAllMarkers function by comparing each cluster with all other clusters within the MCT2 KO dataset. The table lists the significantly enriched marker genes for each cluster that were used for downstream analyses.

**Table S3.** Differentially expressed genes (DEGs) identified in the integrated MCT2 KO and MCT2 CO single-nucleus RNA sequencing dataset. Cluster-specific marker genes were identified using the Seurat FindAllMarkers function by comparing each of the 12 integrated clusters with all other clusters in the integrated dataset. The table lists the significantly enriched marker genes for each cluster that were used for cell type annotation and downstream analyses.

**Table S4.** Differentially expressed genes (DEGs) identified in the MCT2 knockout (KO) versus MCT2 control (CO) comparison for each cell type in the single-nucleus RNA sequencing dataset. DEGs for each cell type were identified using the Seurat FindMarkers function. The accompanying Excel file contains significantly differentially expressed genes for each cell type, with each worksheet corresponding to a cell type for downstream analyses.

**Table S5.** Differentially expressed genes associated with the glycolysis/gluconeogenesis pathway identified across cell clusters in the integrated MCT2 KO and MCT2 CO snRNA-seq dataset. Cluster-specific glycolysis/gluconeogenesis pathway genes were identified using the Seurat FindAllMarkers function. The table lists significantly enriched pathway-associated genes identified in individual cell clusters.

**Table S6.** Differential expression analysis of glycolysis/gluconeogenesis pathway genes between MCT2 KO and MCT2 CO within each cell cluster. Differentially expressed glycolysis/gluconeogenesis pathway genes were identified for each cell cluster using the Seurat FindMarkers function by comparing MCT2 KO and MCT2 CO samples within the same cluster. The table lists significantly differentially expressed pathway-associated genes for each cluster.

**Table S7.** Pseudobulk DESeq2 differential expression analysis of glycolysis/gluconeogenesis genes between MCT2 KO and MCT2 CO across cell clusters. The table lists significantly differentially expressed pathway-associated genes across cell clusters.

**Table S8.** Differentially expressed genes associated with the tricarboxylic acid (TCA) cycle pathway identified across cell clusters in the integrated MCT2 KO and MCT2 CO single-nucleus RNA sequencing dataset. Cluster-specific TCA cycle pathway genes were identified using the Seurat FindAllMarkers function. The table lists significantly enriched TCA cycle pathway-associated genes identified in individual cell clusters.

**Table S9.** Differential expression analysis of tricarboxylic acid (TCA) cycle pathway genes between MCT2 KO and MCT2 CO within each cell cluster**.** Differentially expressed TCA cycle pathway genes were identified for each cell cluster using the Seurat FindMarkers function by comparing MCT2 KO and MCT2 CO samples within the same cluster. The table lists significantly differentially expressed TCA cycle pathway-associated genes for each cluster.

**Table S10.** Pseudobulk DESeq2 differential expression analysis of tricarboxylic acid (TCA) cycle pathway genes between MCT2 KO and MCT2 CO across cell clusters. The table lists significantly differentially expressed pathway-associated genes across cell clusters.

**Table S11.** Differentially expressed genes associated with the oxidative phosphorylation (OXPHOS) pathway identified across cell clusters in the integrated MCT2 KO and MCT2 CO single-nucleus RNA sequencing dataset. Cluster-specific oxidative phosphorylation (OXPHOS) pathway genes were identified using the Seurat FindAllMarkers function. The table lists significantly enriched OXPHOS pathway-associated genes identified in individual cell clusters.

**Table S12.** Differential expression analysis of oxidative phosphorylation (OXPHOS) pathway genes between MCT2 KO and MCT2 CO within each cell cluster. Differentially expressed oxidative phosphorylation (OXPHOS) pathway genes were identified for each cell cluster using the Seurat FindMarkers function by comparing MCT2 KO and MCT2 CO samples within the same cluster. The table lists significantly differentially expressed OXPHOS pathway-associated genes for each cluster.

**Table S13.** Pseudobulk DESeq2 differential expression analysis of oxidative phosphorylation (OXPHOS) pathway genes between MCT2 KO and MCT2 CO across cell clusters. The table lists significantly differentially expressed pathway-associated genes across cell clusters.

**Table S14.** Differentially expressed genes associated with the fatty acid metabolism pathway identified across cell clusters in the integrated MCT2 KO and MCT2 CO single-nucleus RNA sequencing dataset. Cluster-specific fatty acid metabolism pathway genes were identified using the Seurat FindAllMarkers function. The table lists significantly enriched fatty acid metabolism pathway-associated genes identified in individual cell clusters.

**Table S15.** Differential expression analysis of fatty acid metabolism pathway genes between MCT2 KO and MCT2 CO within each cell cluster. Differentially expressed fatty acid metabolism pathway genes were identified for each cell cluster using the Seurat FindMarkers function by comparing MCT2 KO and MCT2 CO samples within the same cluster. The table lists significantly differentially expressed fatty acid metabolism pathway-associated genes for each cluster.

**Table S16.** Pseudobulk DESeq2 differential expression analysis of fatty acid metabolism pathway genes between MCT2 KO and MCT2 CO across cell clusters. The table lists significantly differentially expressed pathway-associated genes across cell clusters.

**Table S17.** Differentially expressed macrophage-associated genes identified across cell clusters in the integrated MCT2 KO and MCT2 CO single-nucleus RNA sequencing dataset. Cluster-specific macrophage-associated genes were identified using the Seurat FindAllMarkers function. The table lists significantly enriched macrophage-associated marker genes identified in individual cell clusters.

**Table S18.** Differential expression analysis of macrophage-associated genes between MCT2 KO and MCT2 CO within each cell cluster. Differentially expressed macrophage-associated genes were identified for each cell cluster using the Seurat FindMarkers function by comparing MCT2 KO and MCT2 CO samples within the same cluster. The table lists significantly differentially expressed macrophage-associated genes for each cluster.

**Table S19.** Pseudobulk DESeq2 differential expression analysis of macrophage-associated genes between MCT2 KO and MCT2 CO across cell clusters. The table lists significantly differentially expressed pathway-associated genes across cell clusters.

**Table S20.** Sample-specific CellChat communication networks are inferred from single-nucleus RNA-sequencing data. CellChat was run independently for three control and three knockout samples. Each worksheet contains the predicted cell-cell communication network for one biological sample, including interacting source and target cell populations and their inferred signaling interactions.

**Table S21.** Summary of nuclei and UMI information before and after each quality control filtering for the MCT2 CO, MCT2 KO, and integrated single-nucleus RNA sequencing datasets. The table summarizes the number of nuclei before and after each quality control filtering and preprocessing, as well as the final number of nuclei retained for downstream analyses. The table also summarizes the UMI information before and after each quality control filtering and preprocessing, and the final number of UMIs retained for downstream analyses.

**Table S22.** Top 15 differentially expressed genes for each cluster extracted from Table S3. The 15 most significantly enriched marker genes for each cluster were selected from Table S3 and used to facilitate cell type annotation of the 12 clusters identified in the integrated MCT2 knockout (KO) and MCT2 control (CO) single-nucleus RNA sequencing dataset.

**Table S23.** Selected marker genes used for cell type annotation of the integrated MCT2 knockout (KO) and MCT2 control (CO) single-nucleus RNA sequencing dataset. Representative marker genes were selected for each of the 12 clusters based on cluster-specific differentially expressed genes identified in Table S3 and established cell type markers reported in the literature. These marker genes were used to assign biological identities to the integrated cell clusters.

**Table S24.** KEGG pathway over-representation analysis of differentially expressed genes identified in the MCT2 knockout (KO) versus MCT2 control (CO) comparison for selected cell clusters. Differentially expressed genes identified using the Seurat FindMarkers function were analyzed with clusterProfiler (*enrichKEGG*) to identify significantly enriched KEGG pathways.

**Table S25.** Gene set enrichment analysis (GSEA) of KEGG pathways using ranked gene expression changes from the MCT2 knockout (KO) versus MCT2 control (CO) comparison for selected cell clusters. Differential gene expression results for each cell cluster were ranked by $\log_2$ fold change and analyzed using clusterProfiler (*gseKEGG*).

**Table S26.** Pseudobulk differential gene expression analysis between MCT2 knockout (KO) and MCT2 control (CO) for each cell type in the single-nucleus RNA sequencing dataset. Gene expression counts from individual nuclei were aggregated by biological replicate within each cell type, and differential expression analysis was performed using DESeq2. The accompanying Excel file contains differential expression results for each cell type, with each worksheet corresponding to one of the 12 annotated cell clusters.

**Table S27.** Gene set enrichment analysis (GSEA) of KEGG pathways based on pseudobulk differential expression results for each cell type. Ranked pseudobulk differential expression results generated by DESeq2 were analyzed using clusterProfiler (*gseKEGG*) to identify significantly enriched KEGG pathways for each annotated cell type. The accompanying Excel file lists the enriched KEGG pathways for each cell type, with each worksheet corresponding to one of the annotated cell clusters.
